# Antitumor Effect of Fluoxetine on Chronic Stress-Promoted Lung Cancer Growth via Suppressing Kynurenine Pathway and Enhancing Cellular Immunity

**DOI:** 10.3389/fphar.2021.685898

**Published:** 2021-08-03

**Authors:** Zhen Yang, Zhuman Li, Zhijun Guo, Yu Ren, Ting Zhou, Zhijun Xiao, Jingjing Duan, Chuangchuang Han, Yuanchi Cheng, Feng Xu

**Affiliations:** ^1^ Department of Central Laboratory, Fengxian Hospital, Southern Medical University, Shanghai, China; ^2^ School of Pharmaceutical Sciences, Southern Medical University, Guangzhou, China; ^3^ Department of Pharmacy, Fengxian Hospital, Southern Medical University, Shanghai, China; ^4^ Department of Neurosurgery, Sixth People’s Hospital South Campus, Shanghai Jiaotong University, Shanghai, China

**Keywords:** fluoxetine, chronic unpredictable mild stress, antitumor effect, kynurenine pathway, cellular immunity

## Abstract

**Background:** Chronic stress promotes cancer growth. Antidepressant fluoxetine (FLX) is usually prescribed for cancer patients with comorbid depression. FLX displays inhibition on cancer cell proliferation, however, the *in vivo* activity has not been investigated.

**Methods:** We explored the antitumor effect of FLX in subcutaneous transplanted lung cancer cells in a tumor-bearing mouse model. Fifty-six C57BL/6 mice were randomly divided into group A (blank control), group B (tumor-bearing control), group C (tumor-bearing + FLX), group D (CUMS control), group E (CUMS + FLX), group F (tumor-bearing + CUMS), and group G (tumor-bearing + CUMS + FLX). 5-HT, tryptophane (Trp), kynurenine, IFN-γ, TNF-α, IL-1α, IL-1β, IL-2, IL-4, IL-6, IL-10, IL-17A levels were measured by ELISA. T helper (Th), cytotoxic T (Tc) and regulatory T cells (Tregs) subtype were measured by flow cytometry. The antitumor effects of FLX were evaluated by tumor weight. The expression of kynurenine pathway related genes TDO, IDO1, IDO2, and apoptosis-related genes caspase1, 3, 4, 5, 7, 12 in tumor tissues were measured by western blotting and qRT-PCR. A549 cells were exposed with FLX (15 μmol/L) and its effect on cell proliferation, migration, and clonal formation were detected. Kynurenine pathway and apoptosis related gene expression were also measured.

**Results:***In vivo*, chronic stress promoted tumor growth in C57BL/6 mice. FLX administration not only significantly reversed chronic unpredictable mild stress (CUMS)-induced reduction of 5-HT and Trp, increment of kynurenine, but increased CD4^+^ Th and CD8^+^ Tc cells, and reduced CD25^+^ FOXP3^+^ Tregs. FLX promoted Th to differentiate into Th1 cells and increased IL-2 and IFN-γ, meanwhile inhibited Th differentiate into Th2 and Th17 cells and decreased the concentrations of IL-4, IL-6, IL-10, and IL-17A. Chronic stress obviously up-regulated IDO1 and IDO2 expression, down-regulated caspase 4, 7, and 12 expression, meanwhile FLX administration reversed this regulation. However, there was no significant change in TDO, caspase 1, 3, 5. Similarly, *in vitro*, FLX administration significantly inhibited the proliferation, migration, and clonal formation of A549 cells and induced cell apoptosis. FLX administration down-regulated the expression of IDO1, IDO2, and up-regulated caspase 4, 5, and 7.

**Conclusion:** Fluoxetine administration could inhibit tumor growth. The inhibition might be via suppressing kynurenine pathway and enhancing cellular immunity.

## Introduction

The occurrence of a tumor is one of the most negative life events. Its poor prognosis, multiple adverse reactions, and high treatment costs can easily lead to depression in patients. About 1/3 of cancer patients are comorbid with depression (Hopwood and Stephens, 2000; Krizanova et al., 2016), which can be treated with antidepressants (Singer et al., 2010). Fluoxetine, a selective serotonin reuptake inhibitor, is a first choice for patients with depression. After fluoxetine treatment, symptoms of depression and the quality of life are significantly improved (Lieb, 2008; Ren et al., 2018). Fortunately, fluoxetine inhibits cancer cells growth including oral, glioma, rectal, and hepatocellular carcinoma cancer cells (Peer et al., 2004; Kannen et al., 2015; Liu et al., 2015; Chen et al., 2019). A few studies indicated that fluoxetine enhance drug sensitivity and reverse multidrug resistance (Zhou et al., 2012; Hsieh et al., 2015). Furthermore, the clinical evidence shows that the long-term use of fluoxetine decreases the risk of lung cancer (Toh et al., 2007).

However, the effects of antidepressants on tumor progression are controversial. Some research suggested fluoxetine could promote tumor progression (Brandes et al., 1992; Steingart and Cotterchio, 1995). The uncertainty of fluoxetine effect on tumor is worthy of further research. Therefore, the purpose of this study was to verify anticancer effect of fluoxetine and possible mechanism in a tumor-bearing mouse model.

Tryptophan, a conditionally essential amino acid, is involved in pain, sleep, and relieves stress response. There are two pathways for tryptophan metabolism: kynurenine pathway and 5-HT pathway. Kynurenine pathway is dependent on Aryl hydrocarbon receptor (AhR) to some extent; its upregulation may lead to cancer progression via down-regulating T-cell phenotype, and then suppressing antitumor immune responses (Li et al., 2011; Adams et al., 2012; Platten et al., 2019). Since our previous study found fluoxetine inhibited the growth and invasiveness of tumor cells *in vitro* and improved the metabolic disorder of amino acids (including tryptophan) *in vivo* induced by chronic stress (Zhou et al., 2012; Ren et al., 2018), we hypothesized that fluoxetine might modulate cellular immunity by suppressing kynurenine pathway. Here we focus on the tryptophan-associated cellular immunity pathway of fluoxetine.

## Materials and Methods

### Chemicals

Fluoxetine was purchased from Sigma (St. Louis, MO, United States). The ELISA kits of 5-HT, Trp, kynurenine, IL-1α, IL-1β, IL-2, IL-4, IL-6, IL-10, IL-17A, IFN-γ, and TNF-α were from Shanghai Jianglai biological technology Co., Ltd. (Shanghai, China). TDO, IDO1, IDO2 antibodies were obtained from Abcam (United States). Caspase-1, Caspase-3, Caspase-4, Caspase-5, Caspase-7, Caspase-12 antibodies were obtained from Immunoway Biotechnology Co., Ltd. (Beijing, China). AhR antibodies were obtained from Proteintech (United States). FITC-conjugated anti-mouse CD3, APC/Cyanine7-conjugated anti-mouse CD4, PE/Cyanine7-conjugated anti-mouse CD8a, APC-conjugated anti-mouse CD25, PE-conjugated anti-mouse Foxp3, APC-conjugated anti-mouse NK-1.1, and PE-conjugated anti-mouse TCR-β antibodies were obtained from Biolegent (California, United States).

### Cell Culture and Animals

Human NSCLC cell line A549 and mice NSCLC cell line LLC were obtained from Shanghai Institutes of Cell Biology (Shanghai, China). Cells were cultured in DMEM medium, supplemented with 10% fetal bovine serum (FBS), 100 μg/ml streptomycin, 100 units/ml penicillin at 5% CO_2_ at 37°C.

Female C57BL/6 mice weighing 18–20 g were bought from the Shanghai Jiesijie laboratory animal technology Co., Ltd. (Animal Quality Certificate: 20180004000898). Mice were housed in the laboratory animal center, East China Normal University, Shanghai (Animal experiment license: SYXK 2010-0094) in a specific pathogen-free (SPF) lab until they were acclimated to their surroundings for 7 days to habituate to the experimenter. The research related animal used has been compiled with all relevant national regulations and institutional policies for the care and use of animals and has been approved by the Animal Research Ethics Committee in Fengxian Hospital, Southern Medical University.

### Behavior Testing–Open-Field Test

The open-field test was conducted in a quiet room. Briefly, the open-field consisted of an opaque plastic box (100 × 100 × 40 cm) divided equally into 20 × 20 cm^2^ squares. Mice were placed in the center of this field, and the number of squares mice moved and standing times were monitored using a ZSZFT Video Analysis System (ZSZRDC science and technology Co., Ltd., China) as indexes of locomotion activity and exploratory behavior, respectively in 5 min. Mouse with behavior scores of >120 or <30 was considered as abnormal and ruled out of the experiment.

### Sucrose Preference Test

Mice were housed individually and exposed to sucrose solution (2% in tap water, Sigma, St Louis, Mo, United States) for 72 h, followed by 18 h of water deprivation and 2 h exposure to two identical bottles, one was filled with 2% sucrose solution and the other was filled with water. The weight of the sucrose solution and water exposed for 2 h was measured. Sucrose preference was defined as the ratio of sucrose weight to total weight (sucrose + water) during the 2 h test, normalized to the body weight of each animal.

### Depression-Like Behavior and Tumor Model

**FIGURE 1:**
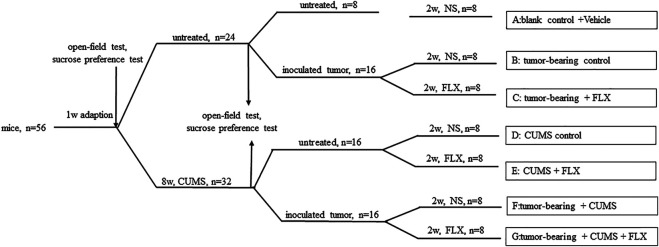
Schematic representation of experimental procedure.

The CUMS-induced depression-like behavior model was established in C57BL/6 mice. The CUMS-induced mice were individually housed in cages for 8 weeks and exposed to the stressors in random order (Table 1). The same stress was not allowed to appear in two consecutive days to avoid prediction. Control mice were fed normally without any stress during this period. 8 weeks later, the open-field test, the sucrose preference experiment and the level of 5-HT were used to verify the success of the depression model. Then, LLC lung cancer cells (1 × 10^6^ cells) suspended in 0.2 ml PBS was subcutaneously inoculated in the right flank of C57BL/6 mice. The entire experimental procedure is shown in Figure 1.

**TABLE 1:** The stressor of CUMS procedure.

Stressor	Duration
restraint stress (activity restriction in a bottle)	1 h
hot water swimming (45°C)	5 min
cold water swimming (4°C)	5 min
horizontal shaking	10 min
wet bedding	24 h
noise interference	10 min
day/night inversion	24 h
cage tilting (45°)	24 h
clip tail (1 cm from the end of the tail)	1 min

### Group and Treatment Protocols

Mice were randomized into seven groups as follows (eight animals each): group A (blank control), group B (tumor-bearing control), group C (tumor-bearing + FLX), group D (CUMS control), group E (CUMS + FLX), group F (tumor-bearing + CUMS), and group G (tumor-bearing + CUMS + FLX). Healthy mice were set as a control without any treatment. After 3 days, fluoxetine (20 mg/kg/day) was administrated via gavage for 2 weeks. Four control groups (groups A, B, D, and F) were given vehicle for 2 weeks. At 24 h after the last administration, all mice were killed. Blood was collected from the inner canthus. Tumors were isolated and weighed.

### ELISA Measurement of 5-HT, Tryptophan, Kynurenine, IL-1α, IL-1β, IL-2, IL-4, IL-6, IL-10, IL-17A, IFN-γ, and TNF-α in Serum

The levels of 5-HT, Tryptophan, kynurenine, IL-1α, IL-1β, IL-2, IL-4, IL-6, IL-10, IL-17A, IFN-γ, and TNF-α in serum were assessed using ELISA kits according to the operating guide.

### Flow Cytometry Analysis of T Cell Testing

To determine the percentages of Th cells, Tc cells, and Treg cells in the total T cell population, the pretreated cells were stained with FITC-conjugated anti-mouse CD3 and treated with fixation and permeabilization regents as described in the manufacturer’s instructions. Then, the cells were stained with antibodies. To determine the percentages of Th in the total CD3^+^ T cell population, the cells were stained with FITC-conjugated anti-mouse CD3, APC/Cyanine7-conjugated anti-mouse CD4, and PE/Cyanine7-conjugated anti-mouse CD8a antibodies. To determine the percentages of regulatory T cells in the total CD4^+^ T cell population, the cells were stained with APC/Cyanine7-conjugated anti-mouse CD4 and APC-conjugated anti-mouse CD25 antibodies and treated with fixation and permeabilization regents. Then, the cells were stained with PE-conjugated anti-mouse Foxp3 antibodies. All the cells were resuspended in 0.2 ml of the staining buffer before being detected by flow cytometry. The data were analyzed with FACS Diva™ software. All antibodies used in the experiment were purchased by Biolegent (California, United States).

### Determination of mRNA Expression

Total RNA was extracted with Trizol according to the protocol (Sangon Biotech, SK1312/BS409, Shanghai, China) and RNA concentration were measured with NanoDrop ND-100 Spectrophotometer (Thermo Scientific, Wilmington, DE, United States). For qRT-PCR, TliRNaseH Plus was used according to the manufacturer’s protocol. The primers sequences are shown in Table 2.

**TABLE 2:** The primer sequences of all genes.

Gene	Forward	Reverse
GAPDH	GAC​ATG​CCG​CCT​GGA​GAA​AC	AGC​CCA​GGA​TGC​CCT​TTA​GT
IDO1	CCC​ACA​CTG​ATC​ACG​GAC​GG	TTG​CGG​GGC​AGC​ACC​TTT​CG
IDO2	CAA​TCC​AGC​CAT​GCC​TGT​GGG​G	TGG​GCT​GCA​CTT​CCT​CCA​GAG​T
Caspase 4	ACG​CAT​GTT​CCG​TTA​CCT​GAA	CAC​CAT​GTG​CCA​TGA​GTA​CCA
Caspase 7	AAG​ACG​GAG​TTG​ACG​CCA​AG	CCG​CAG​AGG​CAT​TTC​TCT​TC
Caspase 12	AGA​CAG​AGT​TAA​TGC​AGT​TTG​CT	TTC​ACC​CCA​CAT​TCC​TTC​C
AhR	ACA​TAC​GCC​GGT​AGG​AAG​AGA	GGT​CAG​CTC​TGT​ATT​GAG​GC

### Western Blotting Analysis of TDO, IDO1, IDO2, AhR, and Cleaved-Caspase Proteins

The tumor tissues and cells were collected and sonicated in lysis buffer on ice. The samples were centrifuged and proteins was quantified by BCA protein assay kit. Total protein (20 μg) from each sample was run on SDS-PAGE and transferred to PVDF membranes. The blots were steeped with 5% fat-free milk in Tris-buffered saline-Tween 20 (TBST) for 4 h, followed by incubation with primary antibody overnight at 4°C. All antibodies were diluted with 5% fat-free milk in TBST buffer (dilution rate: TDO, IDO1, IDO2, and AhR were 1:1,000, cleaved-caspases and β-actin were 1:2,000). The blots were washed with TBST buffer three times (10 min each time), and then labeled with secondary antibody at room temperature for 2 h respectively. Finally, membranes were visualized using ECL detection reagents and analyzed by ImageJ software.

### Cell Viability Assay by CCK-8

The vitro experiments were divided into two groups as follows: control group and fluoxetine group. A549 Cells (5,000 cells per well) were plated into 96-well plates, then treated with fluoxetine for 72 h. Cell viability was assayed using CCK-8 according to the manufacturer’s instructions. The OD value at 450 nm was detected and recorded with GloMaxMuiti+(Promega, E8032, United States).

### Clone Formation Assay

Five hundred cells were seeded in DMEM with 10% FBS on 60 mm plates, and treated with fluoxetine for 12 h, then cultured for 7 days or 10 days. The number of the clones (P50 cells) was assessed by counting under a microscope.

### Cell Migration Assay

Cells were seeded into 6-well plates and treated with fluoxetine for 72 h. The cells were collected and seeded at 0.1 ml (5×10^4^ cells/well) into transwell upper chambers with DMEM. The bottom chambers were filled with 0.6 ml DMEM with 10% FBS as a chemoattractant. After 8 h or 24 h, non-migratory cells were carefully removed with a cotton swab.

### Flow Cytometry Analysis of Apoptosis Assay

A549 Cells were seeded into 6-well plates, treated as above for 24 h/48 h, then collected and washed with PBS twice, stained with Annexin V-FITC and PI (556419, 556421, BD Biosciences). Samples were analyzed on Flow cytometry (BD, FACS Canto, United States). Annexin V-FITC positive and PI negative, Annexin V-FITC positive and PI positive were considered as apoptotic cells.

### Statistical Analysis

All analyses were performed using the SPSS 25.0. Data were expressed as mean ± SD. Statistical comparisons between the two groups were performed using the two independent-samples *t-test* and the inter group were performed using the One-way analysis of variance (*ANOVA*). (*two-tailed*) *p* < 0.05 was considered to be statistically significant.

## Results

### Behavioral and Biochemical Testing

**FIGURE 2:**
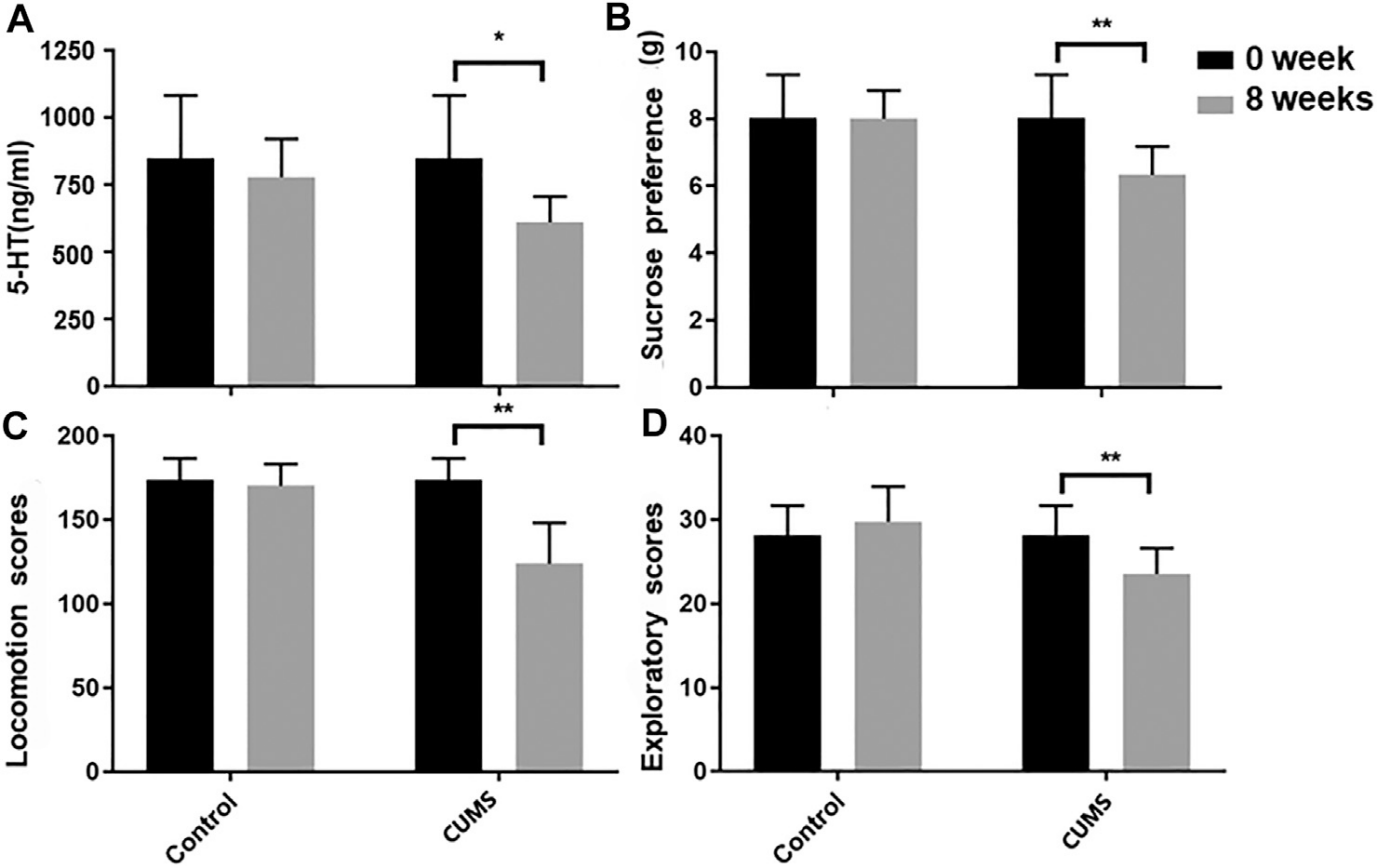
Biochemical test **(A)**, sucrose preference **(B)** and open-field **(C,D)** before and after model establishment. Data were expressed as mean ± SD.

An open-field behavioral test, sucrose preference test, and biochemical test revealed significant differences between two groups after 8 weeks of chronic stress treatment. At the end of 8 weeks of CUMS, the locomotion and exploratory scores, sucrose preference, and 5-HT levels are decreased in CUMS model group mice compared to the baseline. No significant change occurred over time in the normal control group mice (Figure 2). All results confirmed that CUMS successfully induce depression-like behavior in mice.

### CUMS Promoted Tumor Progression, Fluoxetine Inhibited Tumor Growth

**FIGURE 3:**
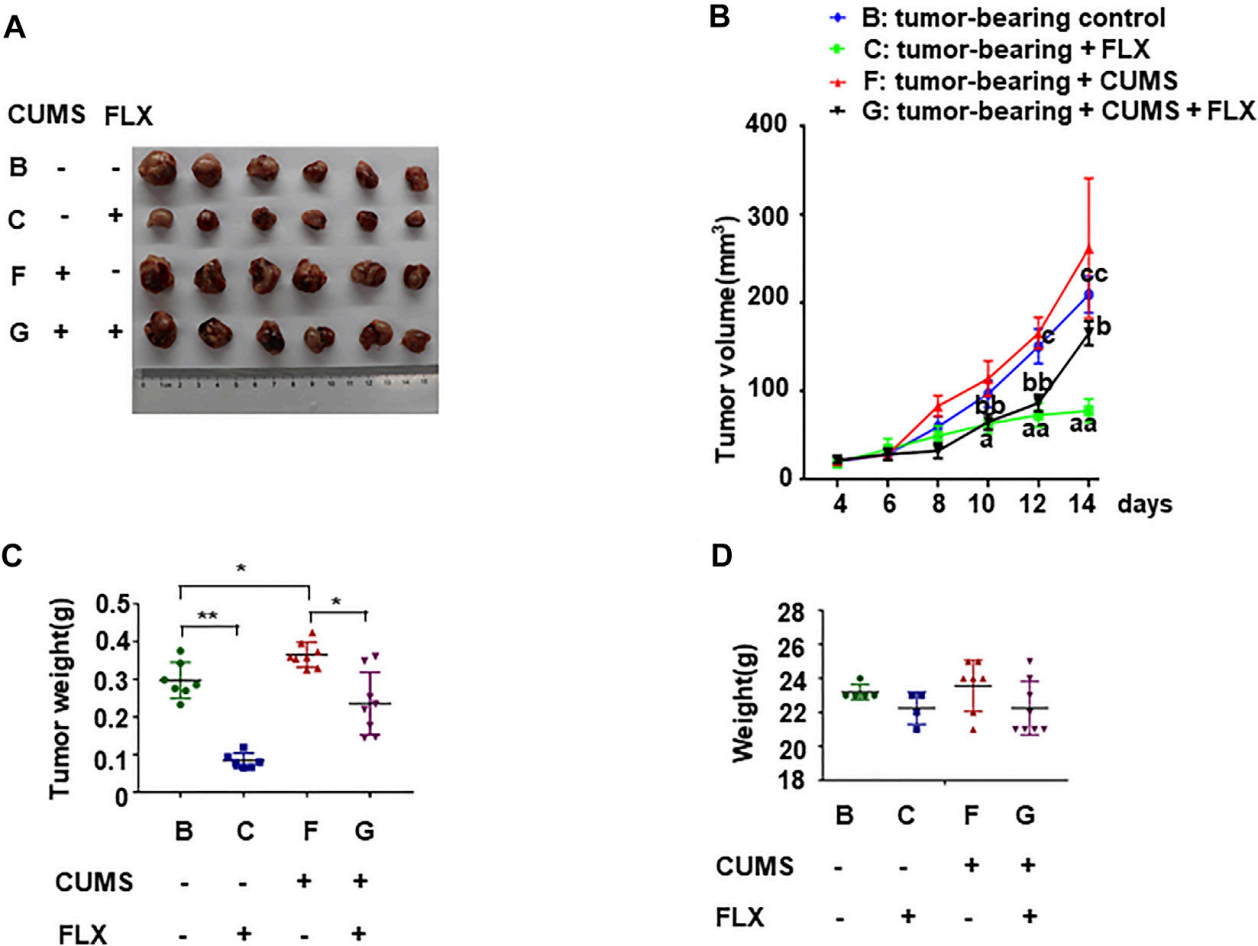
Antitumor of fluoxetine in tumor bearing mice. **(A)** Representative images of tumor tissues in different groups. **(B,C)** Volume and weights of tumor. **(D)** Weights of mice. Data were expressed as mean ± SD. *p < 0.05, **p < 0.01; ^aa^ *p* < 0.01: tumor-bearing control vs. tumor-bearing + FLX; ^bb^ *p* < 0.01: tumor-bearing + CUMS vs. tumor-bearing + CUMS + FLX; ^c^ *p* < 0.05, ^cc^ *p* < 0.01: tumor-bearing control vs. tumor-bearing + CUMS.

Tumor weight and body weight of all tumor-bearing mice were examined. The tumor volume in group B (tumor-bearing control) was significantly smaller than that of group F (tumor-bearing + CUMS). The result indicated that CUMS promoted tumor growth. The tumor volume in group B (tumor-bearing control) was significantly larger than that of group C (tumor-bearing + FLX). Meanwhile the tumor volume in group F (tumor-bearing + CUMS) was significantly larger than that of group G (tumor-bearing + CUMS + FLX), suggesting fluoxetine could inhibit tumor growth in tumor both under stress and without stress (Figures 3A–C). However, there was no difference of the body weight in these four groups (Figure 3D).

### Fluoxetine Increased the Ratio of Tryptophan/Kynurenine

**FIGURE 4:**
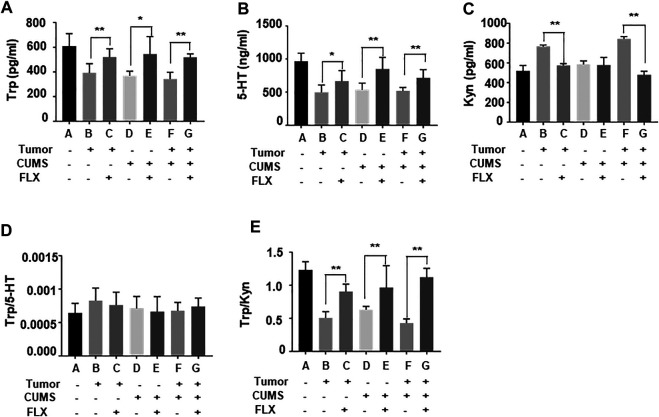
Tryptophan **(A)**, 5-HT **(B)**, kynurenine **(C)**, **(D)** Trp/5-HT and **(E)** Trp/kyn blood levels in mice. Data were expressed as mean ± SD. *p < 0.05, **p < 0.01.

As shown in Figure 4, tryptophan and 5-HT levels in group D (CUMS control), E (CUMS + FLX), F (tumor-bearing + CUMS), G (tumor-bearing + CUMS + FLX) were lower than those of group A (blank control). However, after fluoxetine administration, tryptophan and 5-HT levels in group C (tumor-bearing + FLX), E (CUMS + FLX) and G (tumor-bearing + CUMS + FLX) were higher than those of group B (tumor-bearing control), D (CUMS control) and F (tumor-bearing + CUMS), respectively (Figures 4A,B). Meanwhile, kynurenine levels in group C (tumor-bearing + FLX) and G (tumor-bearing + CUMS + FLX) were lower than those of group B (tumor-bearing control) and F (tumor-bearing + CUMS), respectively (Figure 4C). The ratio of tryptophan/kynurenine was increased after fluoxetine administration (Figure 4E). The data suggested that fluoxetine exerts antitumor effect via increasing the ratio of tryptophan/kynurenine.

### Fluoxetine Enhanced Cellular Immunity *in Vivo*

**FIGURE 5:**
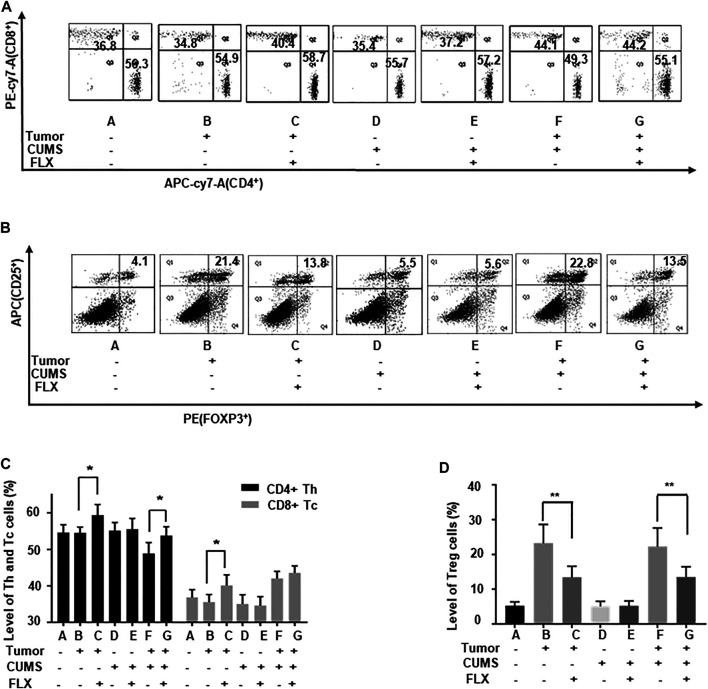
The number of Th, Tc, and Treg cells in the peripheral blood of mice. **(A,C)** The percentages of Th and Tc cells. **(B,D)** The percentages of Treg cells. Data were expressed as mean ± SD. *p < 0.05, **p < 0.01.

The flow cytometry data showed that the numbers of CD4^+^ Th cells and CD8^+^ Tc cells were increased (Figures 5A,C), while the CD25^+^ FOXP3^+^ Treg cells were decreased (Figures 5B,D) after fluoxetine administration. Thus, the results revealed that fluoxetine enhanced Th and Tc cells responses, induced cellular immunity *in vivo*.

### Fluoxetine Modulated the Inflammatory Cytokines

**FIGURE 6:**
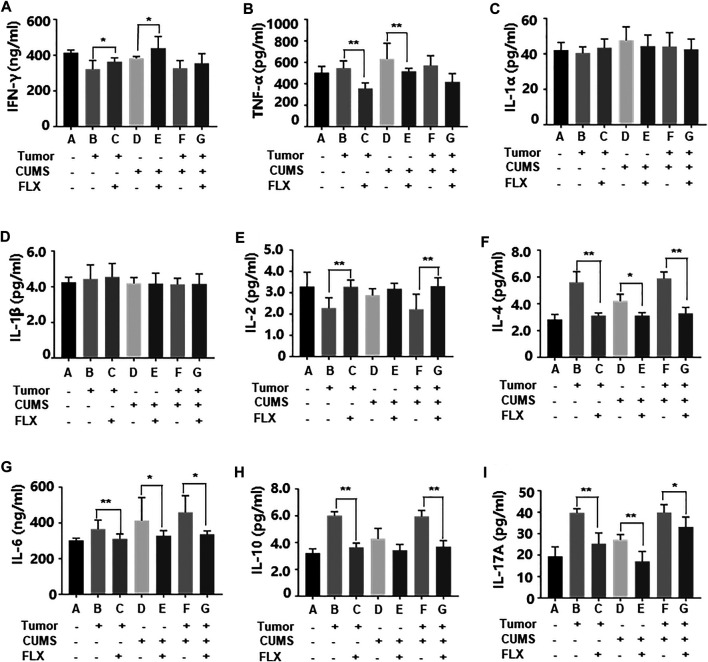
Levels of inflammatory cytokines IFN-γ **(A)**, TNF-α **(B)**, IL-1α **(C)**, IL-1β **(D)**, IL-2 **(E)**, IL-4 **(F)**, IL-6 **(G)**, IL-10 **(H)**, IL-17A (i) in peripheral blood. Data were expressed as mean ± SD. *p < 0.05, **p < 0.01.

As shown in Figure 6, fluoxetine promoted differentiation of CD4^+^ T cells into Th1 cells that increased concentrations of IL-2, IFN-γ; inhibited differentiation of CD4^+^ T cells into Th2 and Th17 cells that decreased concentrations of IL-4, IL-6, IL-10, IL-17A. These data indicated that fluoxetine enhanced cellular immunity, weakened humoral immunity and inflammatory response.

### Fluoxetine Decreased IDO1, IDO2, AhR Expression *in Vivo*

**FIGURE 7:**
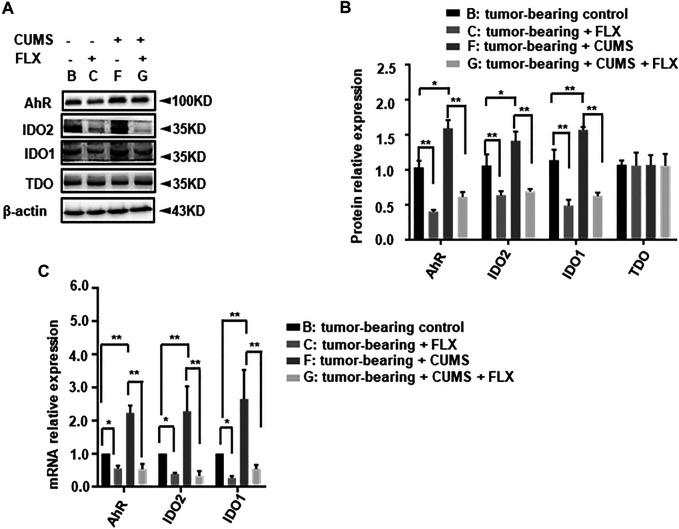
Effect of fluoxetine on kynurenine pathway related enzymes in mice. **(A,B)** Representative and statistical analysis western blotting bands of tumor in mice. **(C)** mRNA relative expression of tumor in mice. Data were expressed as mean ± SD. *p < 0.05, **p < 0.01.

We further investigated the related enzyme expression of kynurenine pathway. The western blot data revealed that CUMS obviously up-regulated IDO1, IDO2 expression, fluoxetine administration reversed this up-regulation (Figure 7). However, there was no significant change in TDO. Meanwhile the AhR expression showed a similar trend as well as IDO. This result indicated that fluoxetine down-regulated IDO1, IDO2, AhR to inhibit kynurenine pathway *in vivo*.

### Fluoxetine Up-Regulated Immune Related Pro-Apoptosis Genes and Executioner Caspase Genes

**FIGURE 8:**
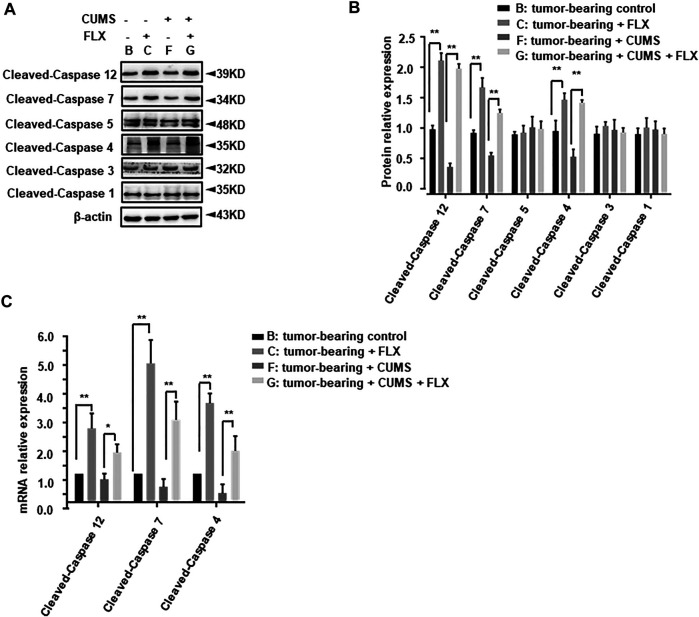
Effect of fluoxetine on immune related pro-apoptosis genes in tumor bearing mice. **(A,B)** Representative and statistical analysis western blotting bands of tumor in mice. **(C)** mRNA relative expression of tumor bearing mice. Data were expressed as mean ± SD. **p < 0.01.

Further, we investigated the key immune related pro-apoptosis genes (cleaved-caspase 1, 4, 5, 12) and executioner caspase genes (cleaved-caspase 3, 7). As shown in Figure 8 **,** cleaved-caspase 4, 7, 12 showed a significant increase in group C (tumor-bearing + FLX) and group G (tumor-bearing + CUMS + FLX) compared with group B (tumor-bearing control) and group F (tumor-bearing + CUMS), respectively. Fluoxetine significantly potentiated the expression of cleaved-caspase 4, 7, and 12 in comparison with the vehicle group (Figure 8). However, there was no significant change in cleaved-caspase 1, 3, or 5. These results indicated the pro-apoptotic and regulated inflammation cytokines activities of fluoxetine administration *in vivo*.

### Fluoxetine Exerts an Anti-Cancer Effect *in Vitro*

**FIGURE 9:**
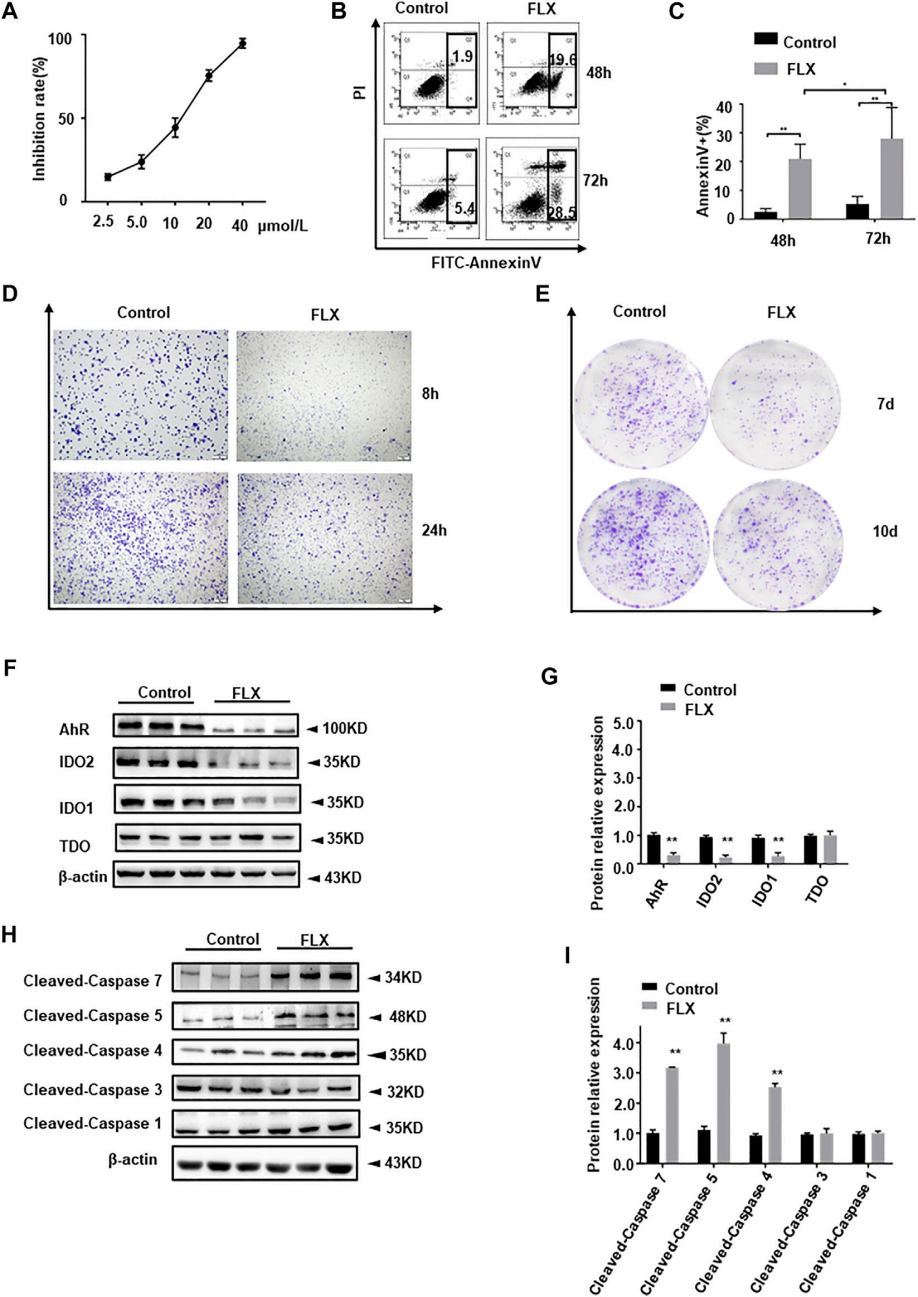
Anticancer effect of fluoxetine in A549 cells. **(A)** Growth-inhibition curves of cells. **(B,C)** Cell apoptosis of A549 cells. **(D)** Cell migration of A549 cells. **(E)** Clone formation of A549 cells. **(F,G)** Representative and statistical analysis western blotting bands of tryptophan metabolic enzymes in A549 cells. **(H,I)** Representative and statistical analysis western blotting bands of apoptosis-related protein in A549 cells. Data were expressed as mean ± SD. *p < 0.05, **p < 0.01.

We verified the antitumor effect of fluoxetine in A549 cells. The growth-inhibition curves showed that the 50% inhibition concentration (IC_50_) of fluoxetine was 15 μmol/L (Figure 9A). Compared with the control group, fluoxetine induced apoptosis and reduced migration and clonal formation (Figures 9B–E). Moreover, fluoxetine down-regulated IDO1, IDO2, and AhR expression and up-regulated immune related pro-apoptosis protein cleaved-caspase 4, 5, and 7 (Figures 9F–I). But there was no significant change in cleaved-caspase 1, 3, or 5. The results of the *in vitro* experiment were consistent with those *in vivo*.

## Discussion

Chronic stress destroys immune homeostasis gradually which results in promoted tumor growth and proliferation (Hilakivi-Clarke and Dickson, 1995; Thaker et al., 2007; Hefner et al., 2014; Zhao et al., 2015). In this study, we confirmed that chronic stress promoted tumor progression and found that fluoxetine had antitumor effects, possibly by suppressing the kynurenine pathway and enhancing cellular immunity.

In the present study, we established a mouse model of depression comorbid with tumor successfully. We verified the anti-tumor effect of fluoxetine in a tumor-bearing mouse model. The tumor weight in the control group was less than that in the CUMS group. Fluoxetine significantly inhibited the tumor growth compared with the corresponding control group. The above results indicate that fluoxetine is an antidepressant drug with anticancer effects.

The immunosuppressive protein IDO is a rate-limiting enzyme that inhibits T-cell proliferation by catabolizing tryptophan into the kynurenine pathway, leading to rapid proliferation of tumors (Boasso et al., 2007; Li et al., 2011). Our preliminary mechanism study showed that fluoxetine significantly inhibited the expressions of IDO1 and IDO2, increased tryptophan and 5-HT, and decreased kynurenine levels. These results suggested that fluoxetine suppressed the kynurenine pathway and promoted tryptophan accumulation. Low levels of tryptophan at the tumor site causes T cells to arrest in the G1 phase of the cell cycle, which may represent an intrinsic immune escape mechanism of tumor cells (Uyttenhove et al., 2003). Tryptophan metabolites such as serotonin and kynurenine play an important role in regulating immune function (Osawa et al., 2011). 5-HT is an important immune regulatory factor, which can enhance the phagocytosis ability and promote the activation of T cells (Askenase et al., 1991; Young and Matthews, 1995; Leon-Ponte et al., 2007). In our result, high levels of 5-HT and low levels of kynurenine may enhance anti-tumor immunity. The immunosuppressive protein IDO is a rate-limiting enzyme that inhibits T-cell proliferation by catabolizing tryptophan into the kynurenine pathway, leading to rapid proliferation of tumors.

With the low level of kynurenine, we noted that the number of Th1 T cells and the IL-2, IFN-γ level were increased in peripheral blood. We also found that the number of Th2 cells and Treg cells were significantly decreased, the levels of IL-4, IL-6, IL-10 and TNF-α were reduced. These data indicated that fluoxetine enhanced cellular immunity, weakened humoral immunity and inflammatory response (McHugh and Shevach, 2002; Kim and Maes, 2003; Workman et al., 2009). In addition, the key immune related pro-apoptosis genes including cleaved-caspase 4, −12 and executioner caspase genes cleaved-caspase 7 were up-regulated compared with corresponding control groups. However, cleaved-caspase 4, 5, and 7 were upregulated in cell assay. Immune related pro-apoptosis genes are involved in cytokine-mediated inflammation and play an auxiliary role in the exogenous apoptotic pathway (Gurung and Kanneganti, 2015). Different activation states of caspases can achieve different cell death phenotypes for significant anti-cancer effects (Nagarajan et al., 2019). It should be noteworthy that there is a close relationship between immune mechanisms and apoptotic regulation control in normal and malignant tissues (Ucker and Levine, 2018).

As in clinical chemotherapy practice, it is routine for anticancer drugs combination. Therefore, we will further study the anticancer drug such as cisplatin combination with fluoxetine in animal experiments study to confirm whether fluoxetine presents a synergistically antitumor effect. In general, our work suggests that more attention should be paid to cancer patients with depression. Fluoxetine may be a promising potential adjunct therapy for cancer patients in the future.

## Conclusion

**FIGURE 10:**
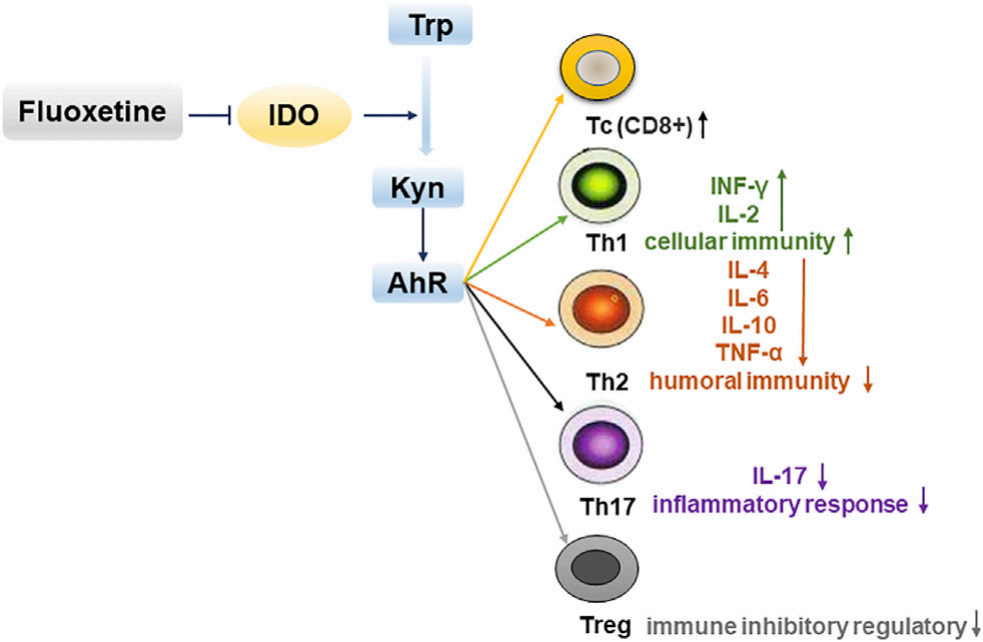
Fluoxetine enhances cellular immunity via down-regulating kynurenine pathway-associated genes IDO1, IDO2, AhR to inhibit tumor progression.

In summary, our results confirmed that depression promoted the growth of cancer. Fluoxetine is antidepressant drug with anticancer effect which may be via suppressing kynurenine pathway and enhancing cellular immunity (Figure 10). These findings suggest that fluoxetine is a promising drug for cancer patients with depression.

## Data Availability

The raw data supporting the conclusion of this article will be made available by the authors, without undue reservation.
