# Supplementary figures and images for: Antitumor Effect of Fluoxetine on Chronic Stress-Promoted Lung Cancer Growth via Suppressing Kynurenine Pathway and Enhancing Cellular Immunity
Source: Front Pharmacol. 2021 Aug 3;12:685898. doi: 10.3389/fphar.2021.685898 (PMC8369900; doi:10.3389/fphar.2021.685898)

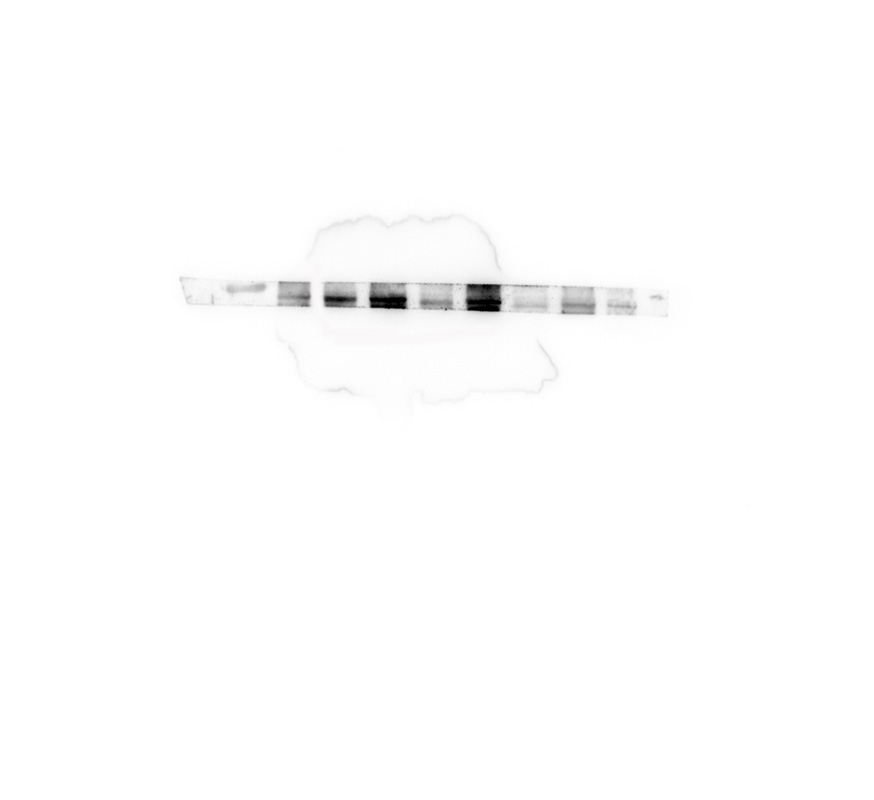

Supplement: Supplementary file 1 [file DataSheet1.ZIP › original source data/FIGERE 7 FIGERE 8Wesrern blotting of tumor/7.1 AhR(left2-5).jpg]

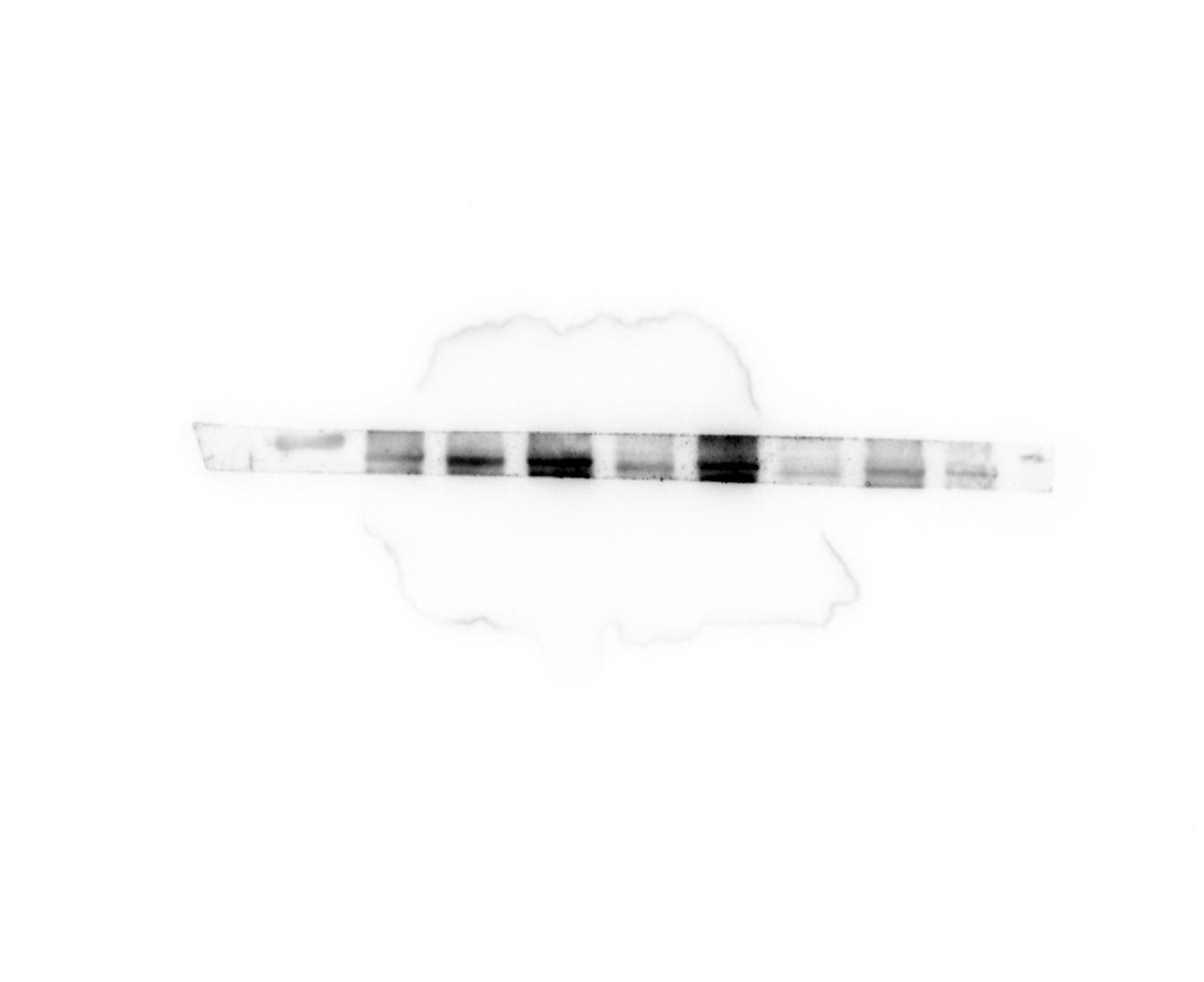

Supplement: Supplementary file 1 [file DataSheet1.ZIP › original source data/FIGERE 7 FIGERE 8Wesrern blotting of tumor/7.2 IDO2(left2-5).Tif]

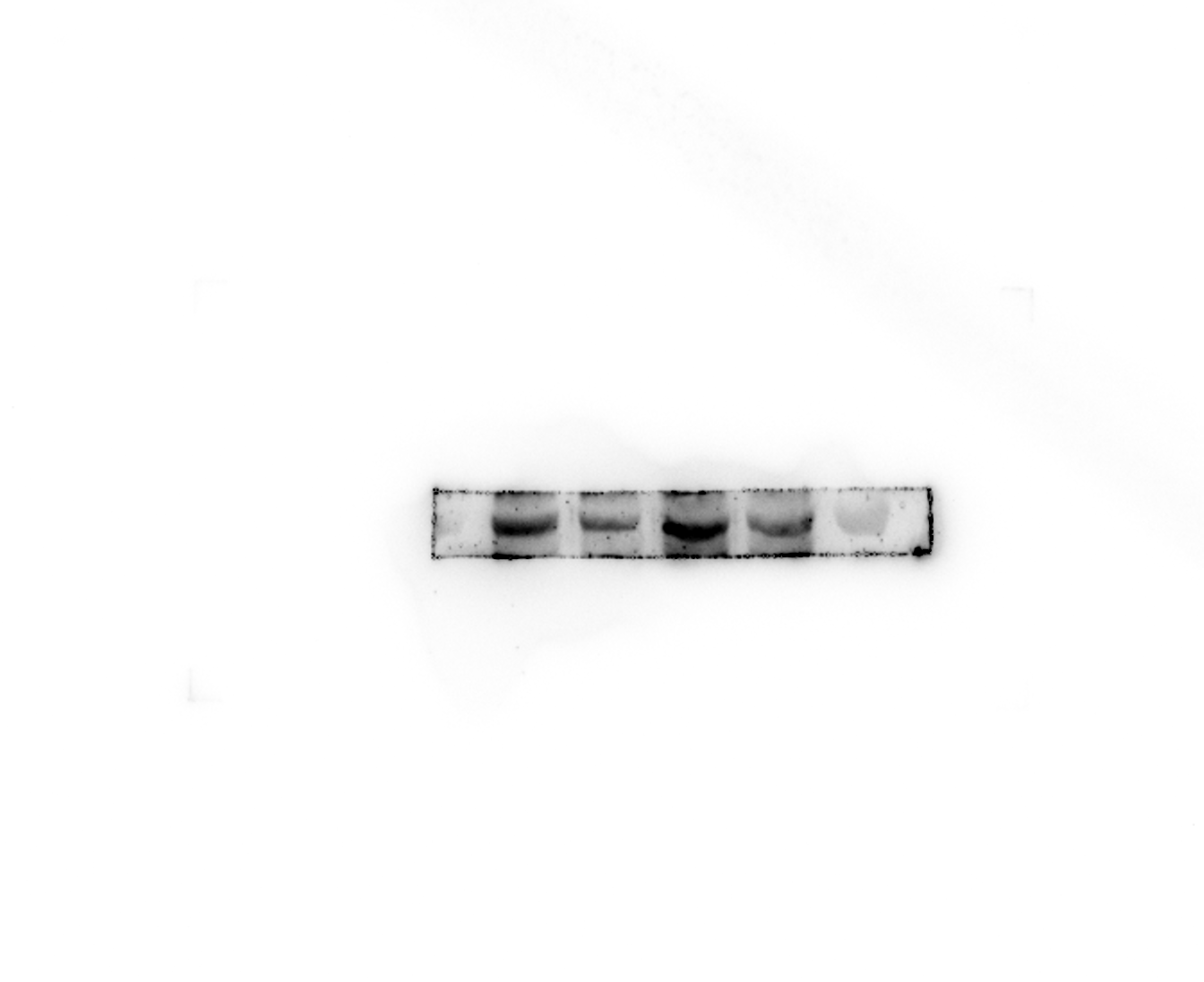

Supplement: Supplementary file 1 [file DataSheet1.ZIP › original source data/FIGERE 7 FIGERE 8Wesrern blotting of tumor/7.3 IDO1.Tif]

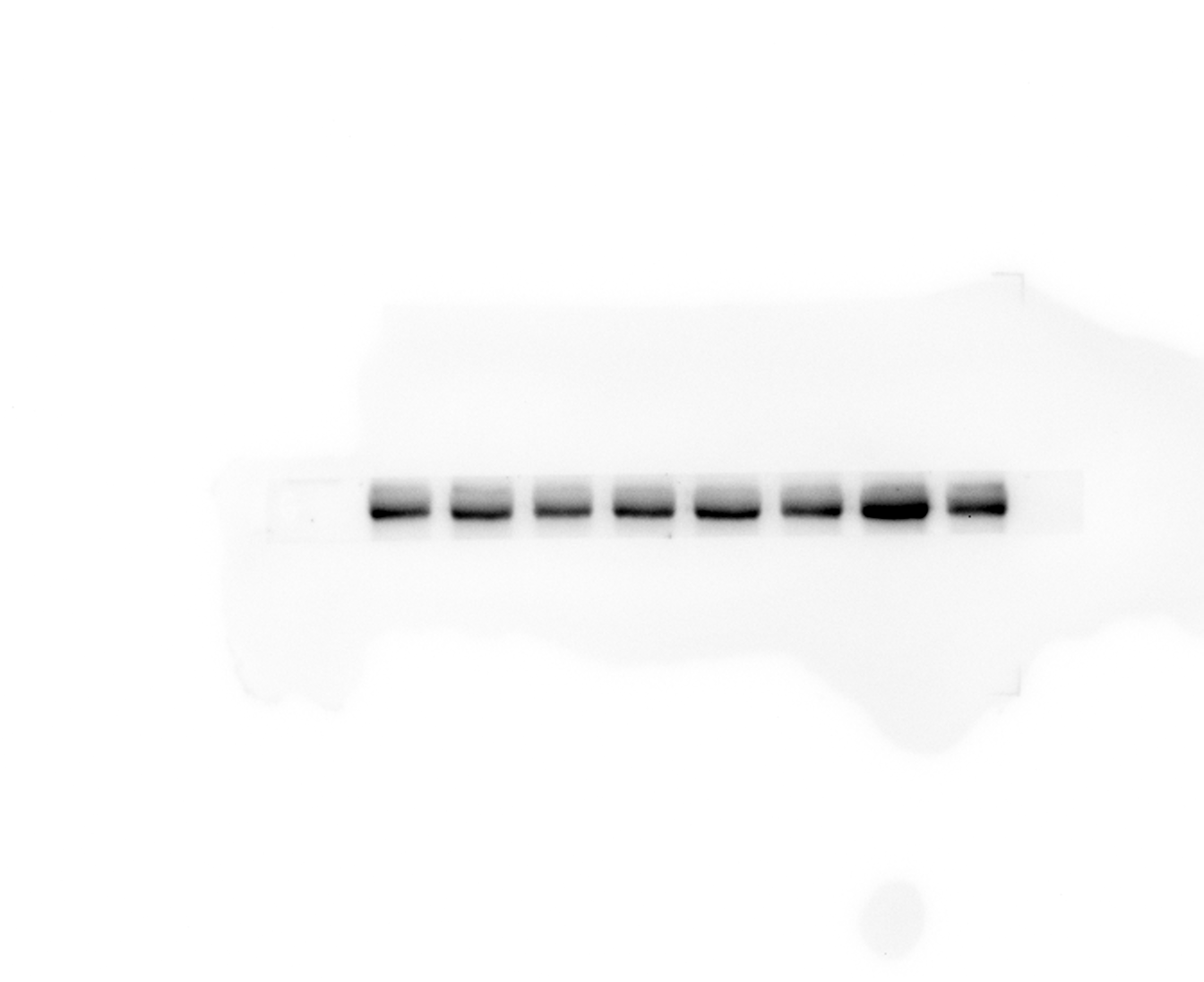

Supplement: Supplementary file 1 [file DataSheet1.ZIP › original source data/FIGERE 7 FIGERE 8Wesrern blotting of tumor/7.4 TDO(left1-4).Tif]

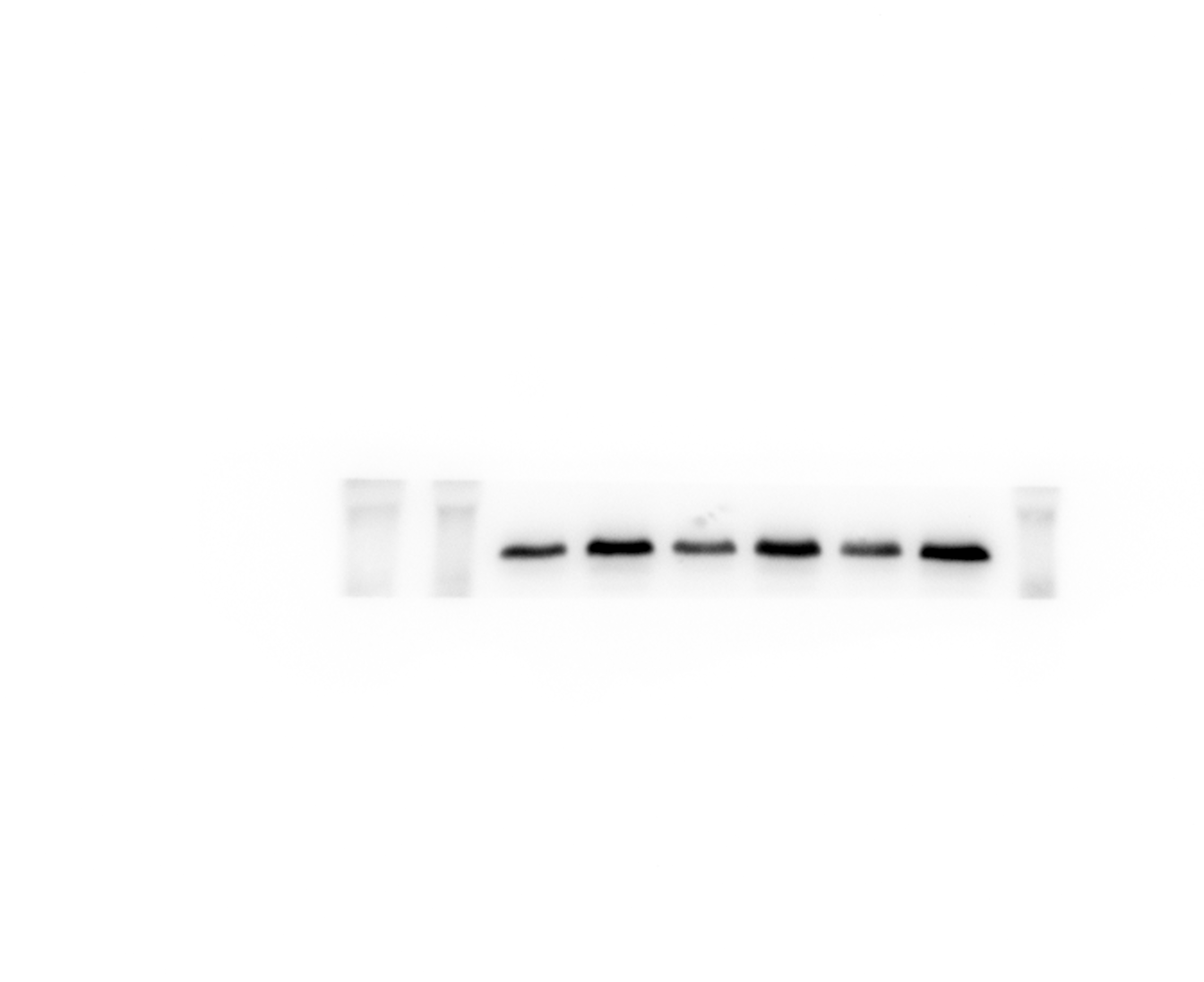

Supplement: Supplementary file 1 [file DataSheet1.ZIP › original source data/FIGERE 7 FIGERE 8Wesrern blotting of tumor/8.1 cleaved-caspase12(left1-4).Tif]

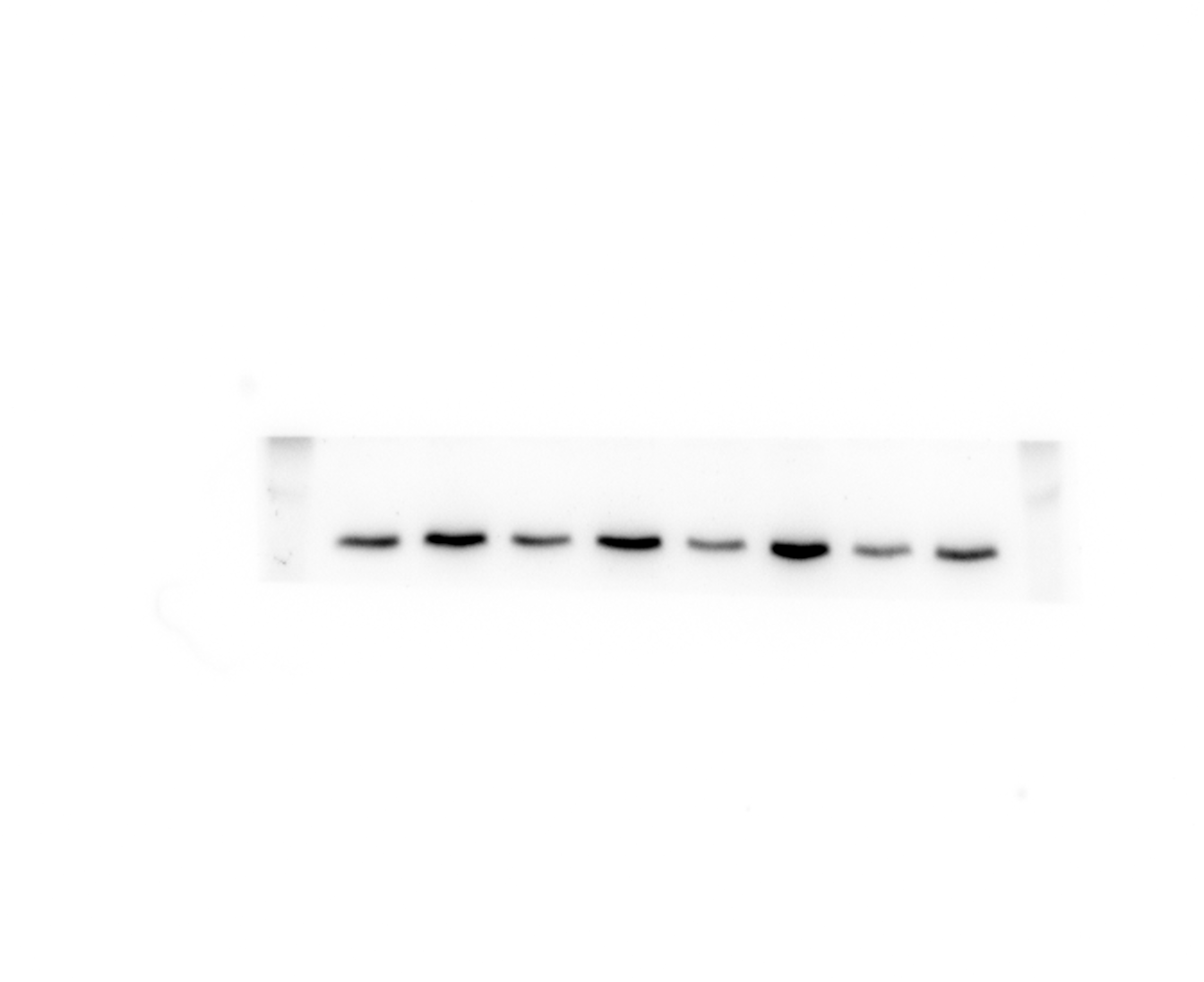

Supplement: Supplementary file 1 [file DataSheet1.ZIP › original source data/FIGERE 7 FIGERE 8Wesrern blotting of tumor/8.2 cleaved-caspase7(left1-4).Tif]

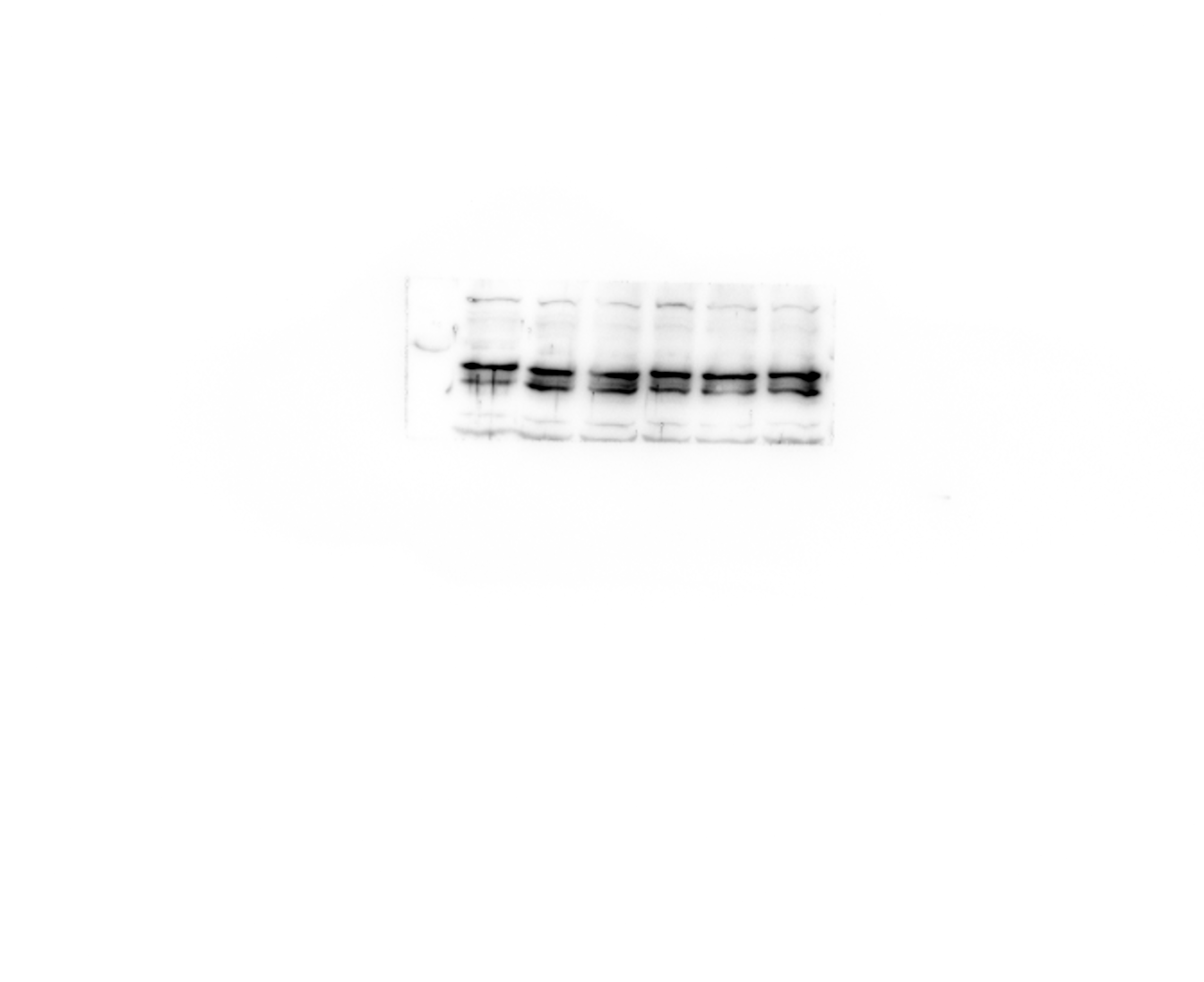

Supplement: Supplementary file 1 [file DataSheet1.ZIP › original source data/FIGERE 7 FIGERE 8Wesrern blotting of tumor/8.3 cleaved-caspase5(left3-6).Tif]

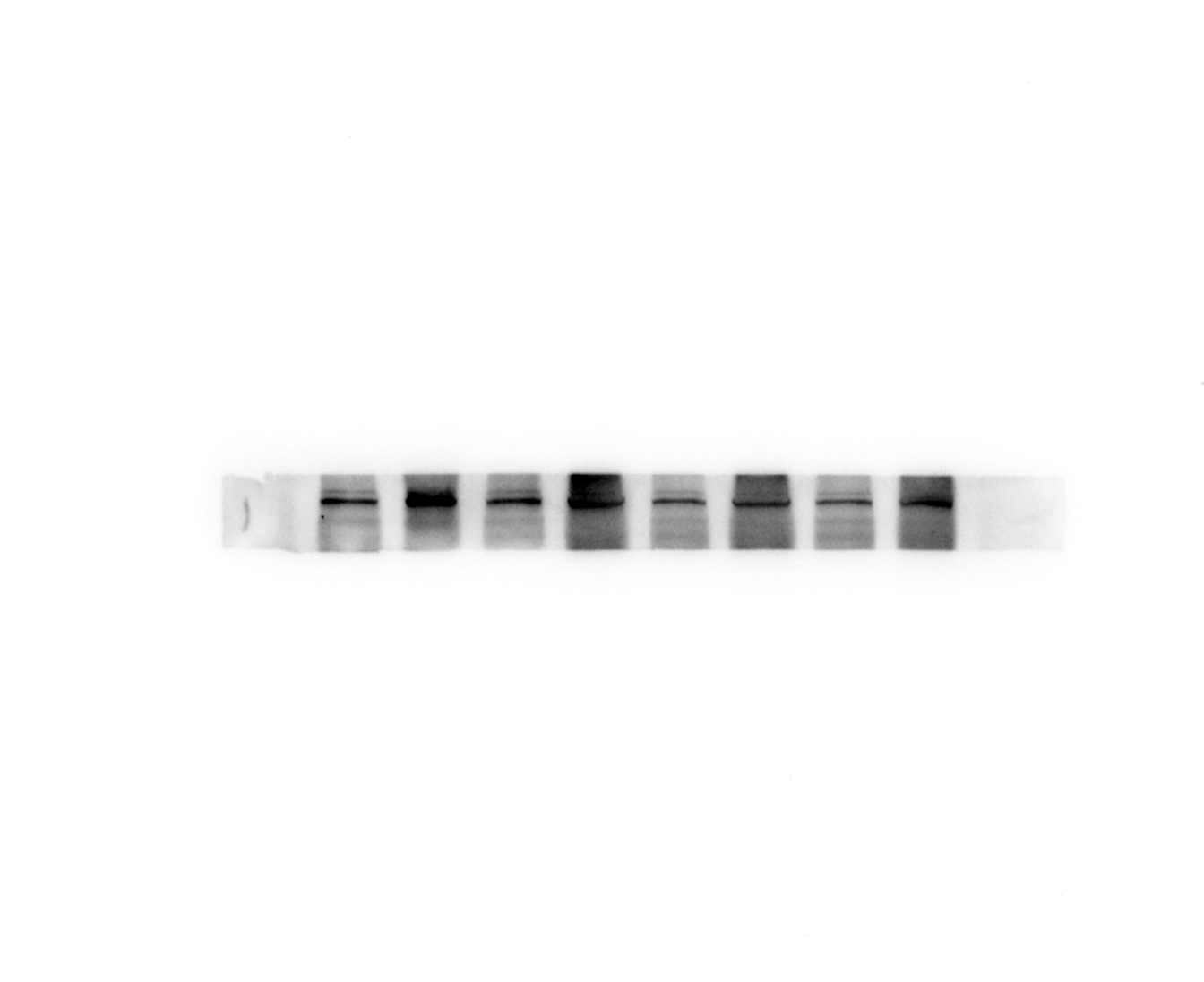

Supplement: Supplementary file 1 [file DataSheet1.ZIP › original source data/FIGERE 7 FIGERE 8Wesrern blotting of tumor/8.4 cleaved-caspase4(left1-4).Tif]

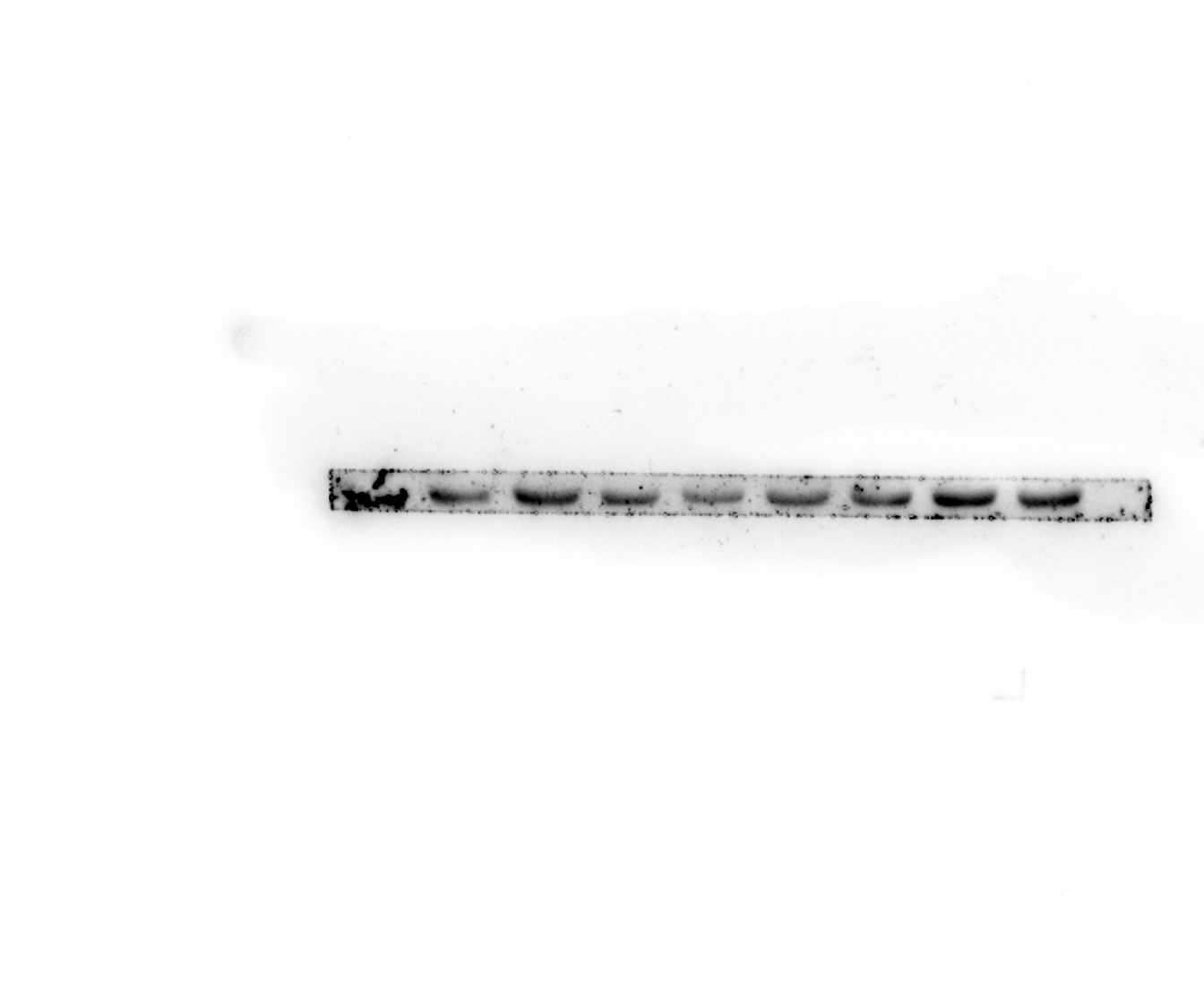

Supplement: Supplementary file 1 [file DataSheet1.ZIP › original source data/FIGERE 7 FIGERE 8Wesrern blotting of tumor/8.5 cleaved-caspase3(left5-8).Tif]

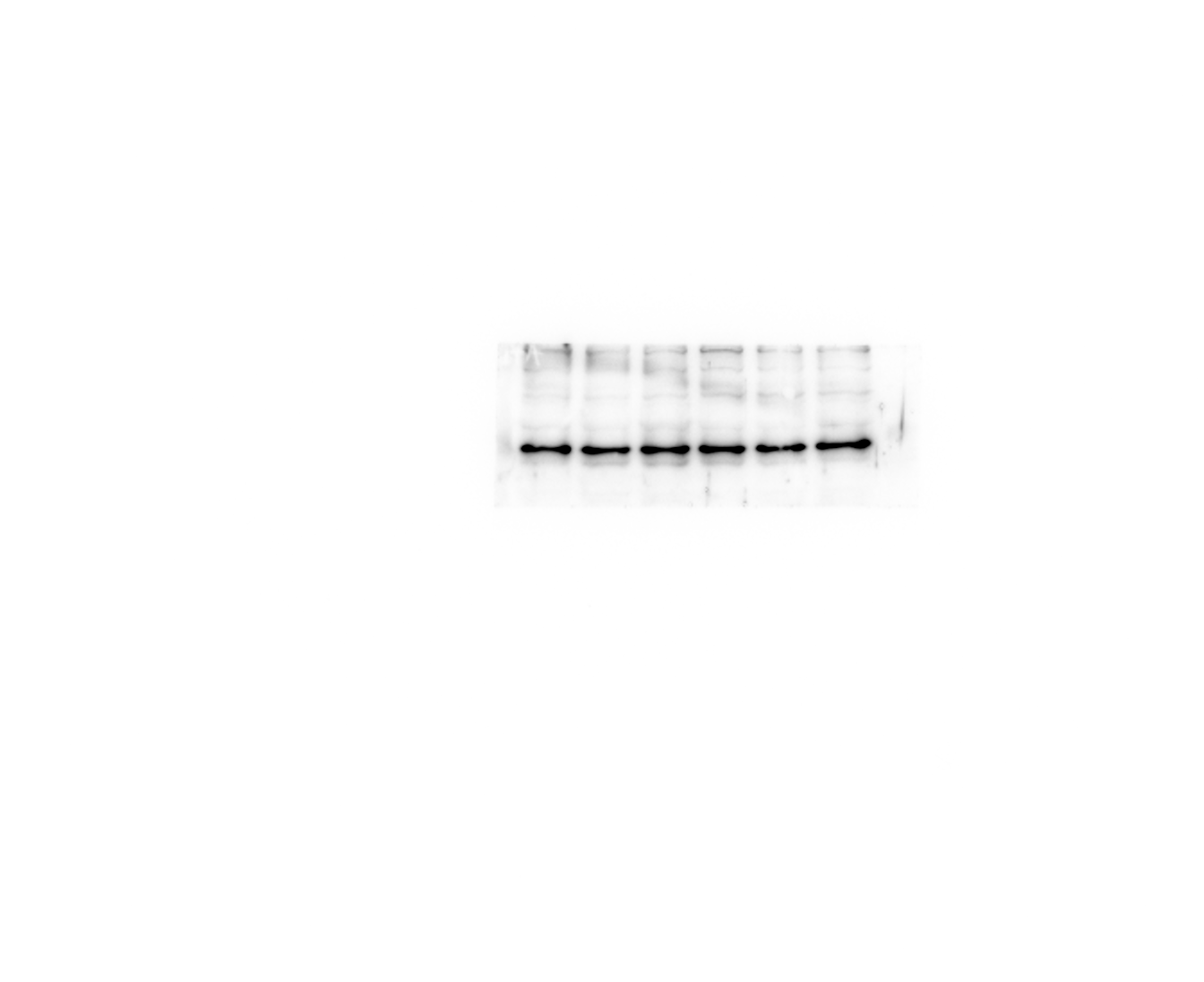

Supplement: Supplementary file 1 [file DataSheet1.ZIP › original source data/FIGERE 7 FIGERE 8Wesrern blotting of tumor/8.6 cleaved-caspase1(left1-4).Tif]

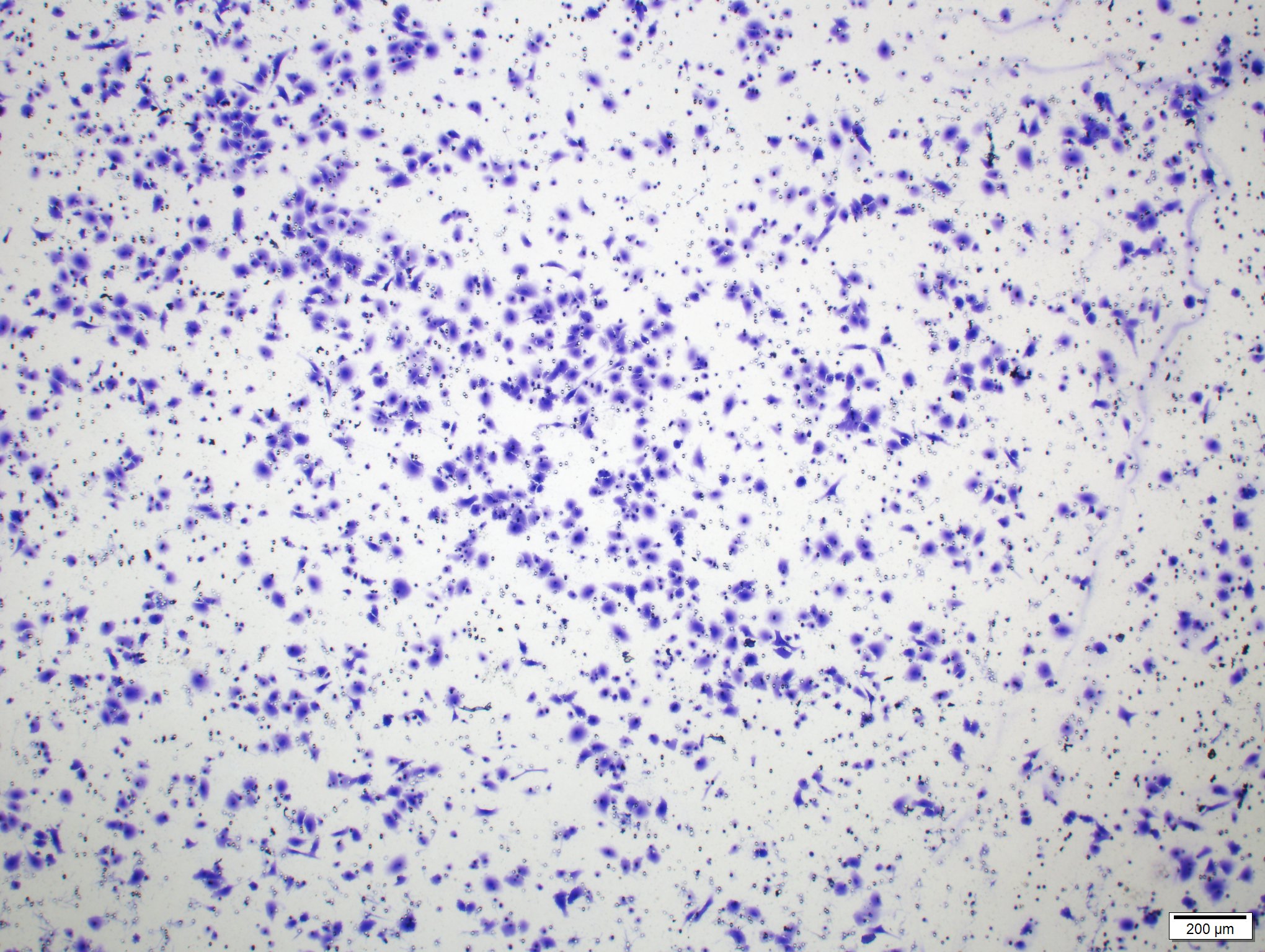

Supplement: Supplementary file 1 [file DataSheet1.ZIP › original source data/FIGERE 9 Anticancer effect of fluoxetine in A549 cells/cell migration/con(24h).jpg]

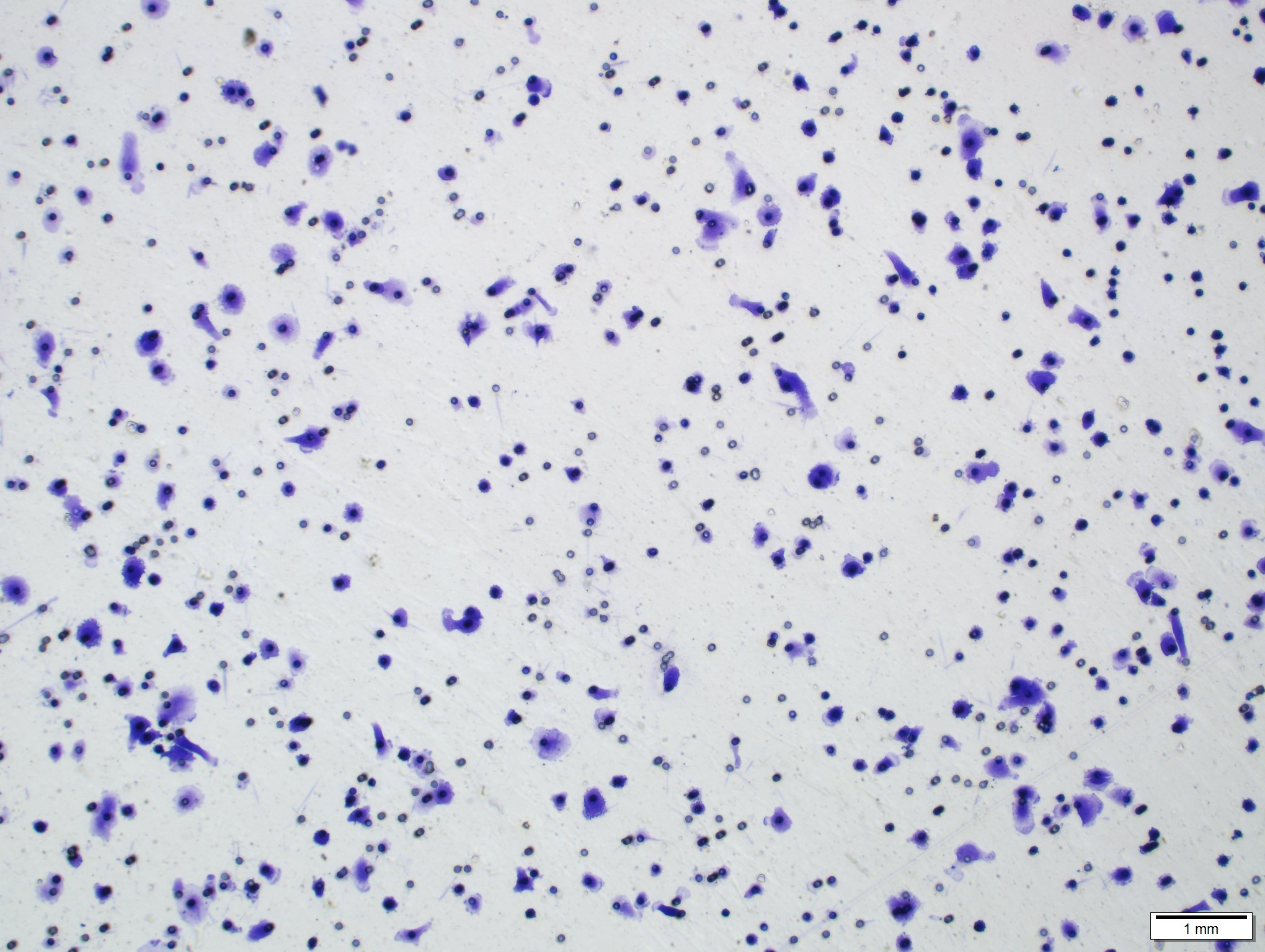

Supplement: Supplementary file 1 [file DataSheet1.ZIP › original source data/FIGERE 9 Anticancer effect of fluoxetine in A549 cells/cell migration/control(8h).jpg]

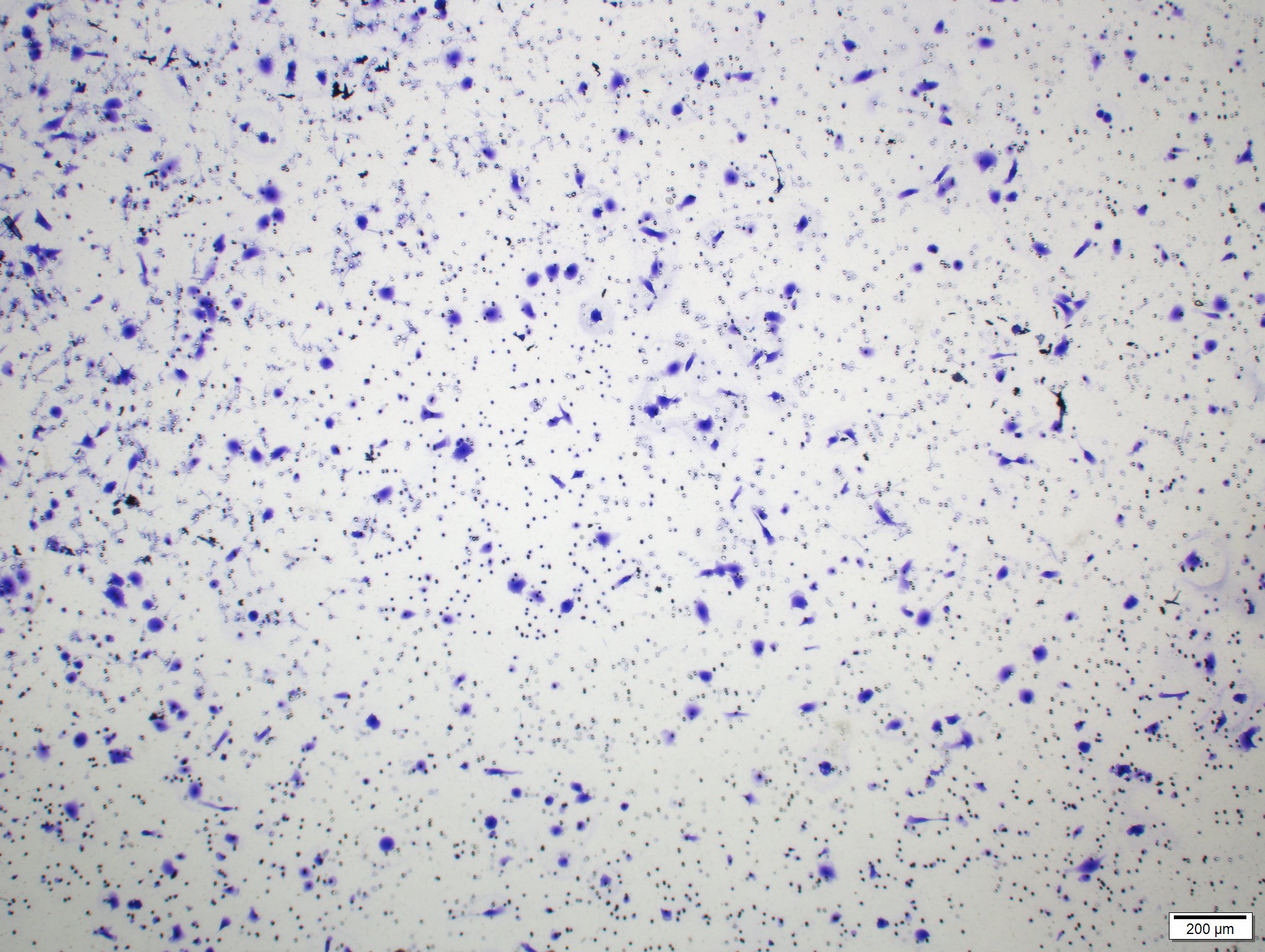

Supplement: Supplementary file 1 [file DataSheet1.ZIP › original source data/FIGERE 9 Anticancer effect of fluoxetine in A549 cells/cell migration/flx(24h).jpg]

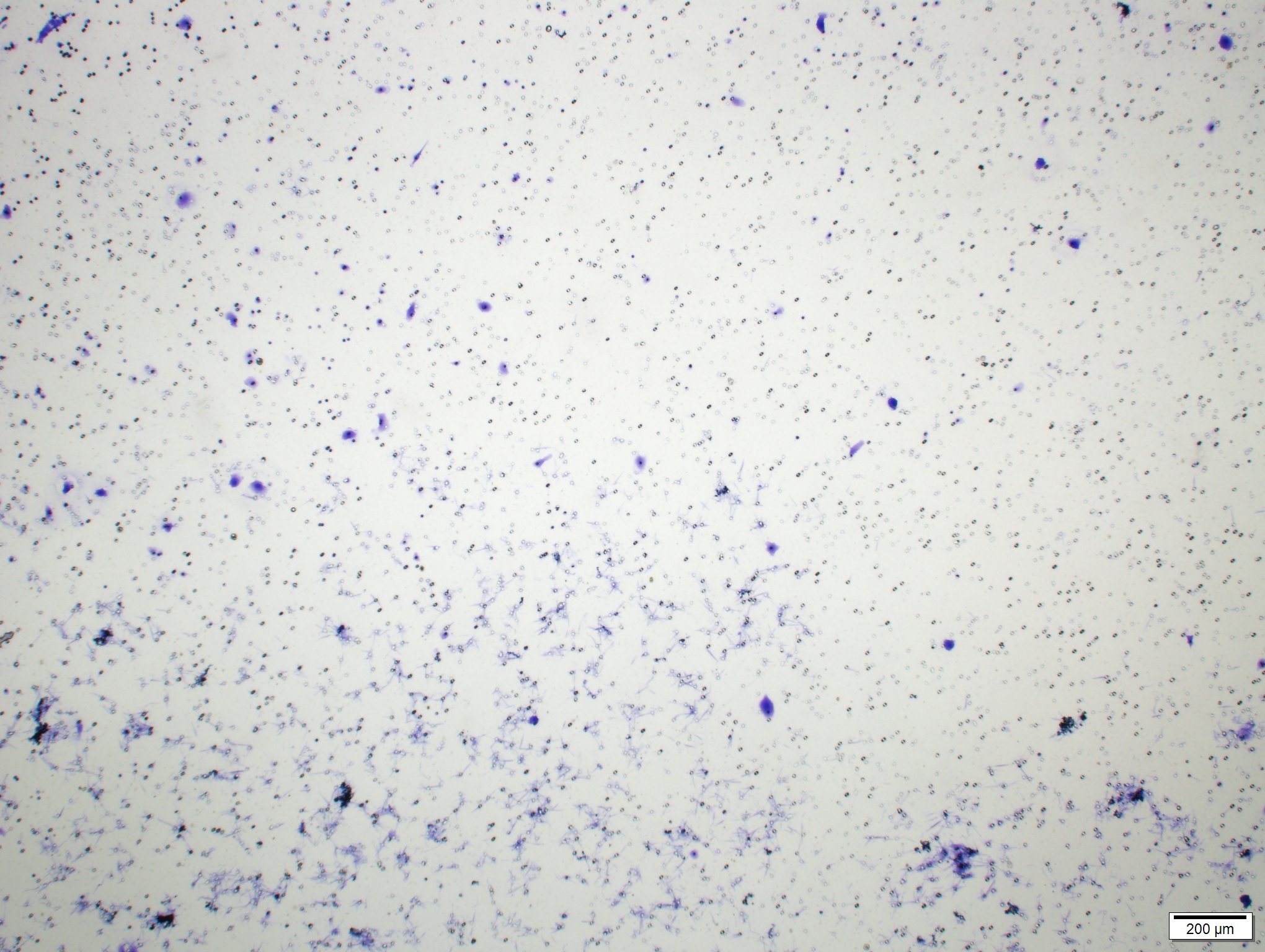

Supplement: Supplementary file 1 [file DataSheet1.ZIP › original source data/FIGERE 9 Anticancer effect of fluoxetine in A549 cells/cell migration/flx(8h).jpg]

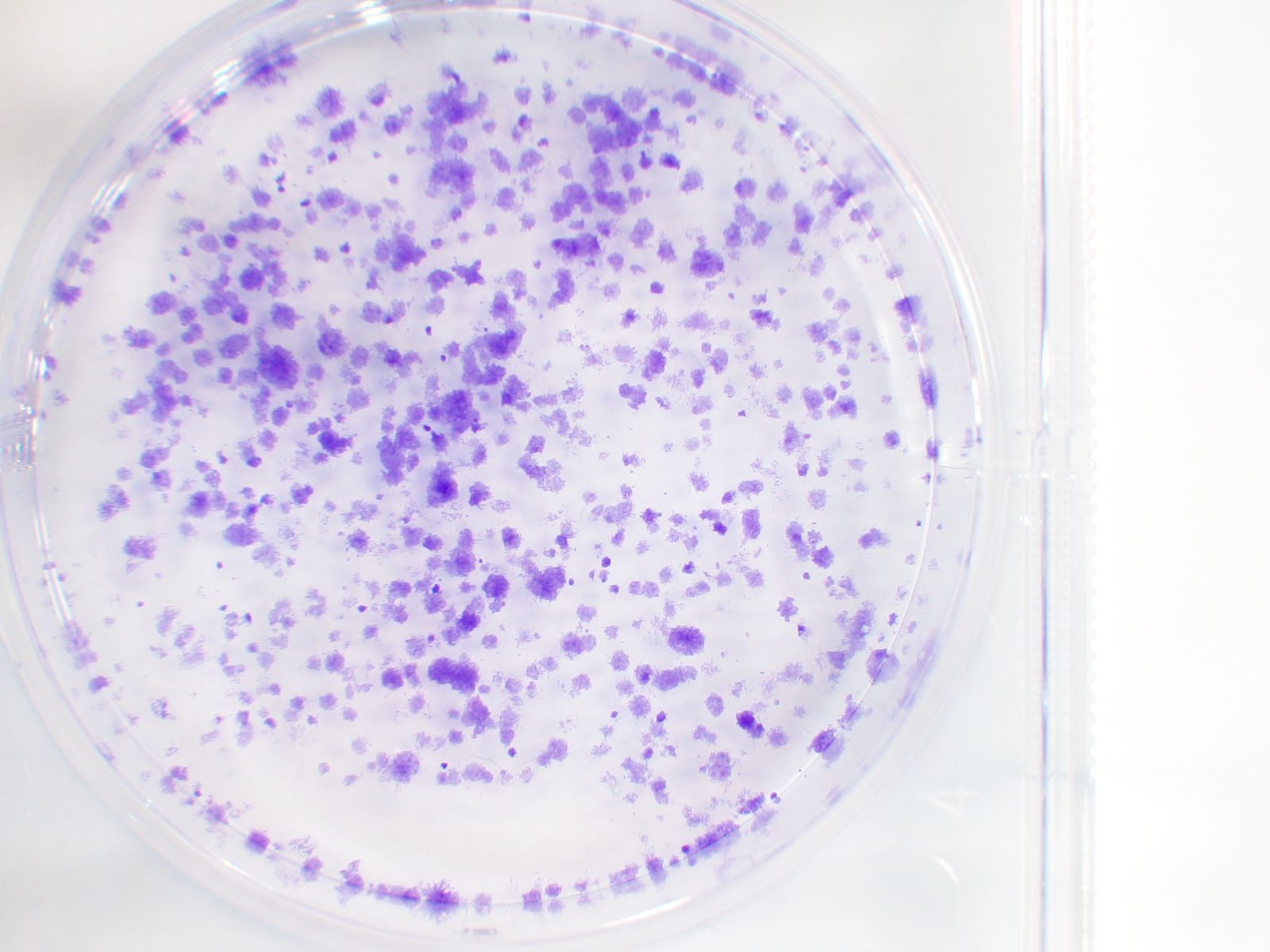

Supplement: Supplementary file 1 [file DataSheet1.ZIP › original source data/FIGERE 9 Anticancer effect of fluoxetine in A549 cells/control(10day).jpg]

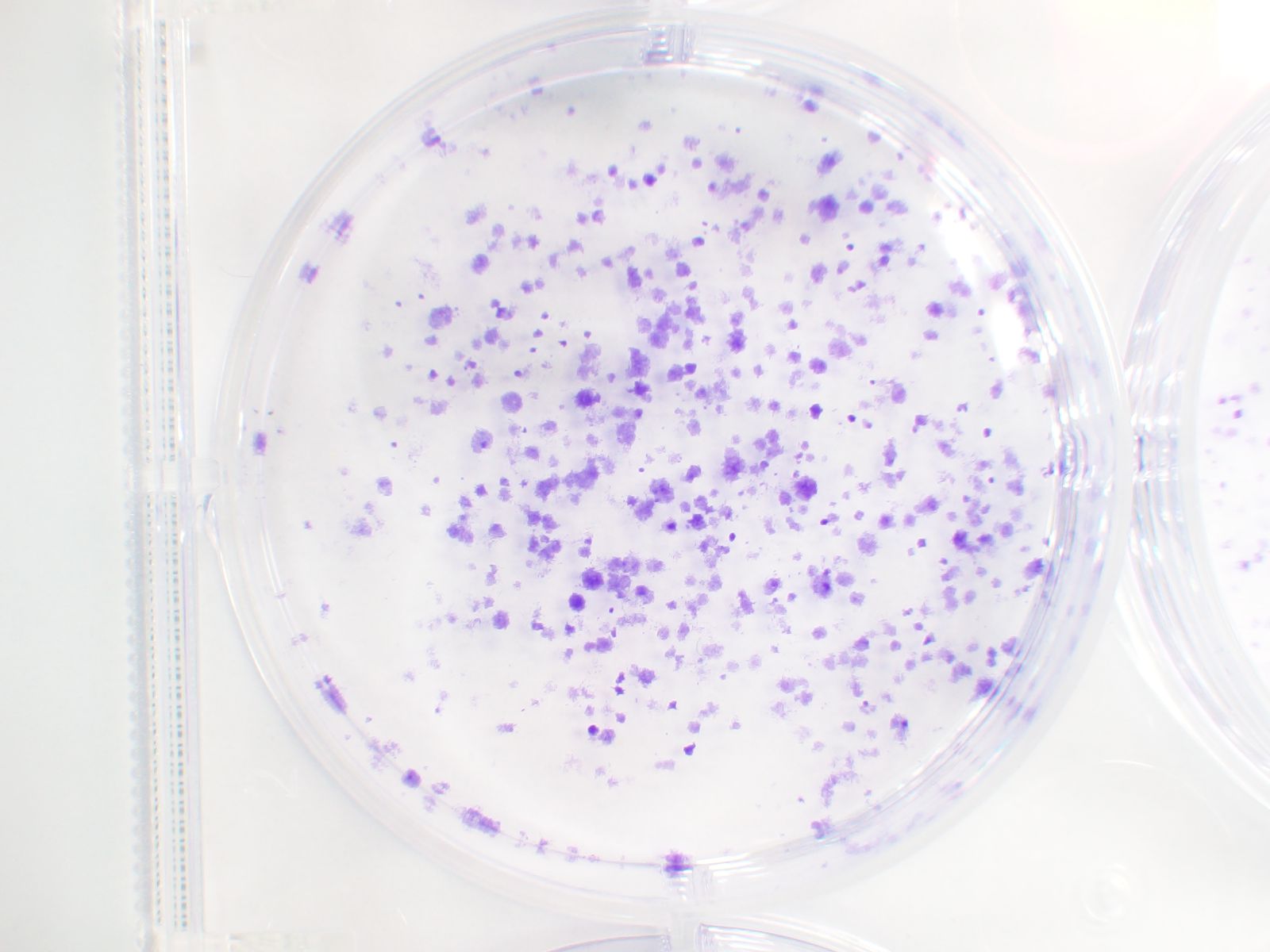

Supplement: Supplementary file 1 [file DataSheet1.ZIP › original source data/FIGERE 9 Anticancer effect of fluoxetine in A549 cells/control(7day).jpg]

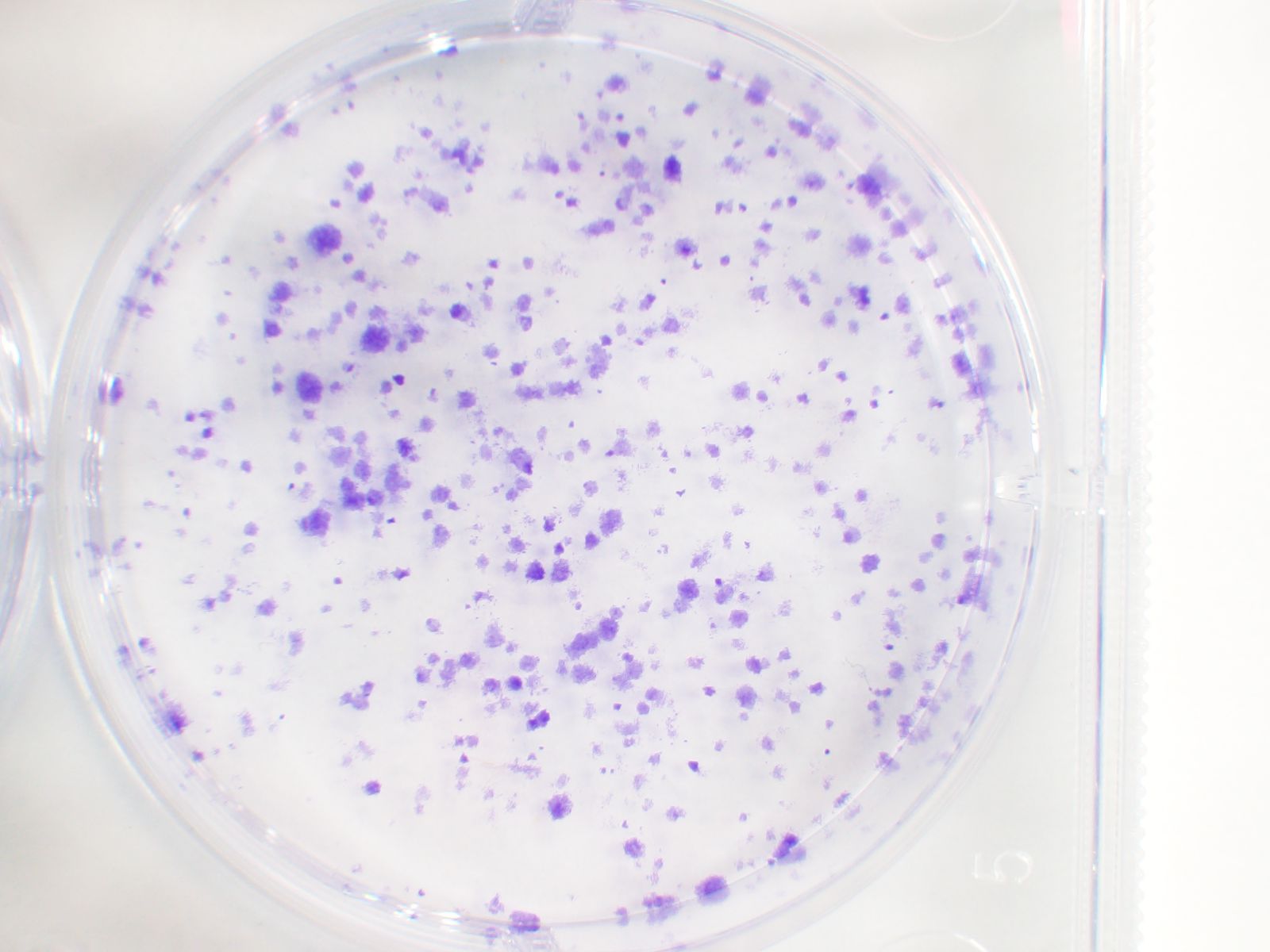

Supplement: Supplementary file 1 [file DataSheet1.ZIP › original source data/FIGERE 9 Anticancer effect of fluoxetine in A549 cells/flx(10day).jpg]

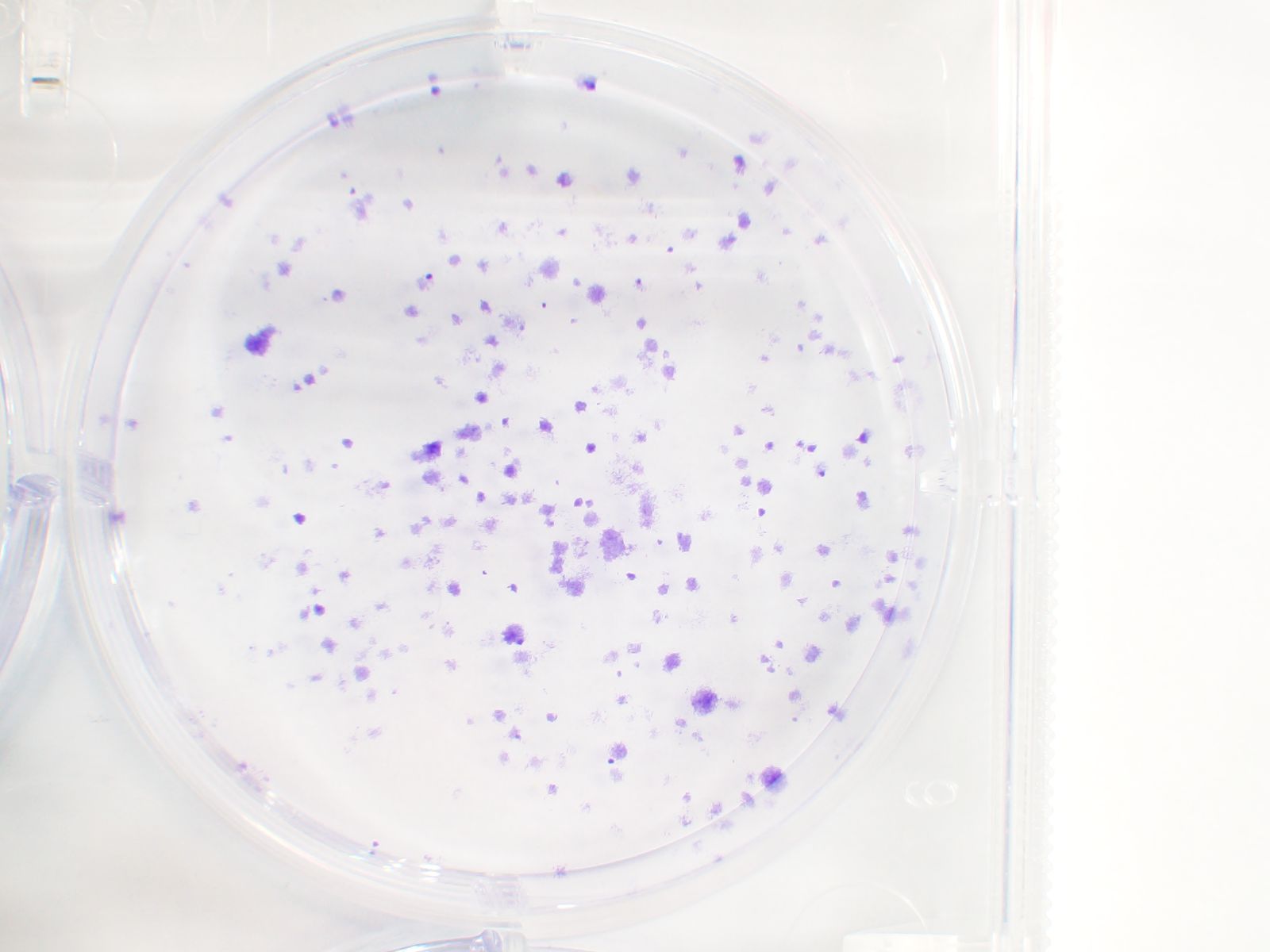

Supplement: Supplementary file 1 [file DataSheet1.ZIP › original source data/FIGERE 9 Anticancer effect of fluoxetine in A549 cells/flx(7day).jpg]

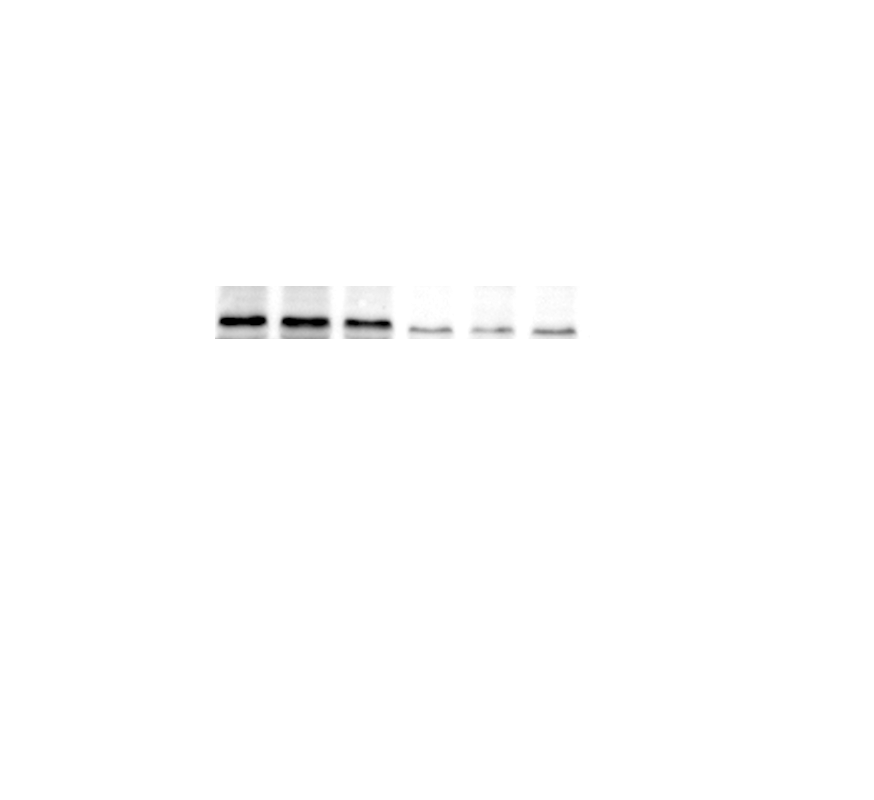

Supplement: Supplementary file 1 [file DataSheet1.ZIP › original source data/FIGERE 9 Anticancer effect of fluoxetine in A549 cells/Wesrern blotting of A549 cell/9f1 AhR.tif]

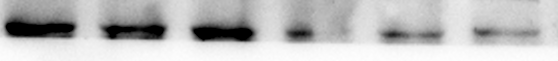

Supplement: Supplementary file 1 [file DataSheet1.ZIP › original source data/FIGERE 9 Anticancer effect of fluoxetine in A549 cells/Wesrern blotting of A549 cell/9f2 IDO2.Tif]

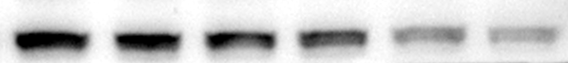

Supplement: Supplementary file 1 [file DataSheet1.ZIP › original source data/FIGERE 9 Anticancer effect of fluoxetine in A549 cells/Wesrern blotting of A549 cell/9f3 IDO1.Tif]

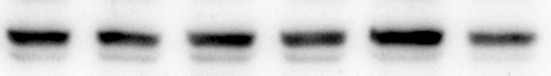

Supplement: Supplementary file 1 [file DataSheet1.ZIP › original source data/FIGERE 9 Anticancer effect of fluoxetine in A549 cells/Wesrern blotting of A549 cell/9f4 TDO.Tif]

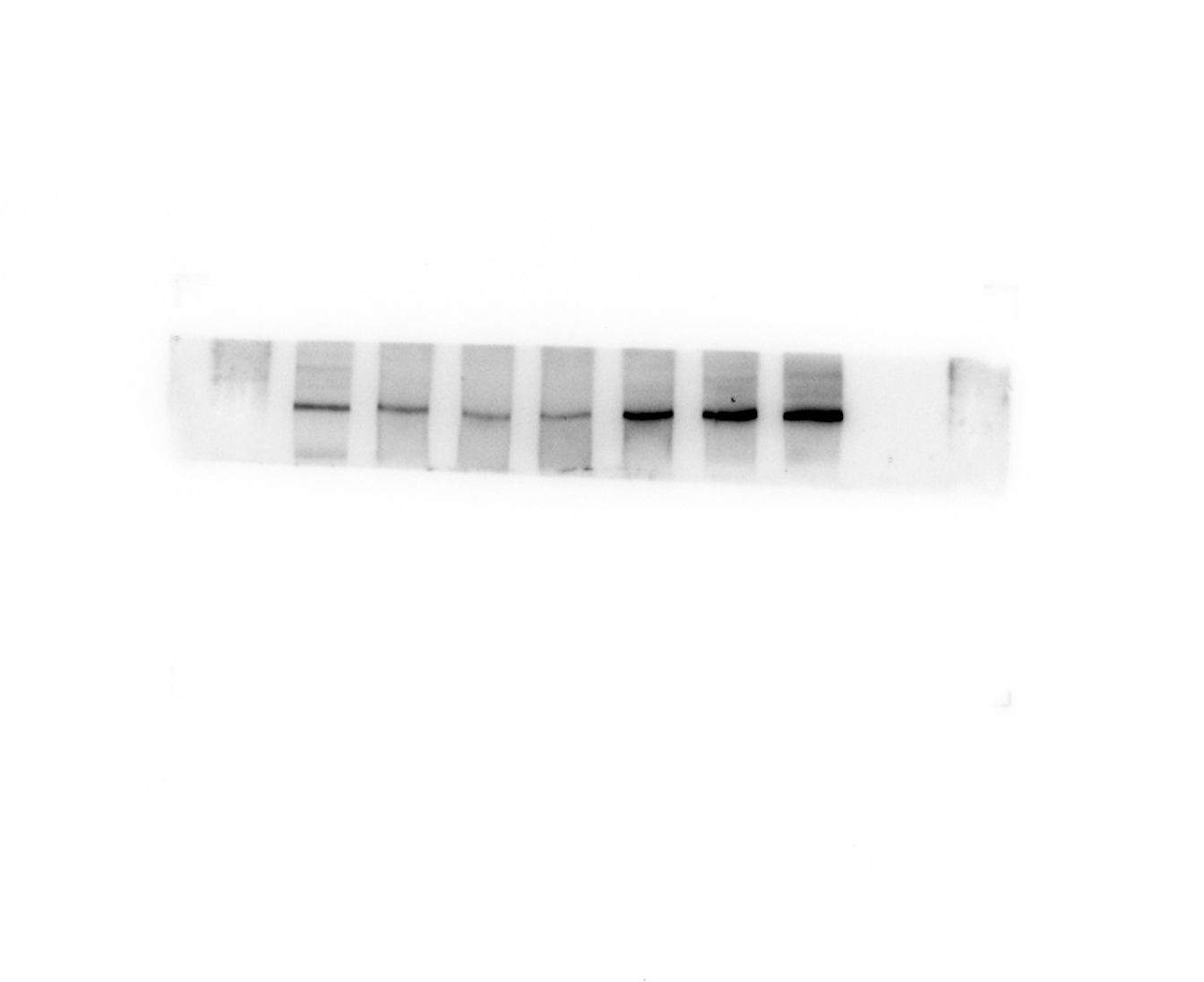

Supplement: Supplementary file 1 [file DataSheet1.ZIP › original source data/FIGERE 9 Anticancer effect of fluoxetine in A549 cells/Wesrern blotting of A549 cell/9h1 cleaved-caspase7.Tif]

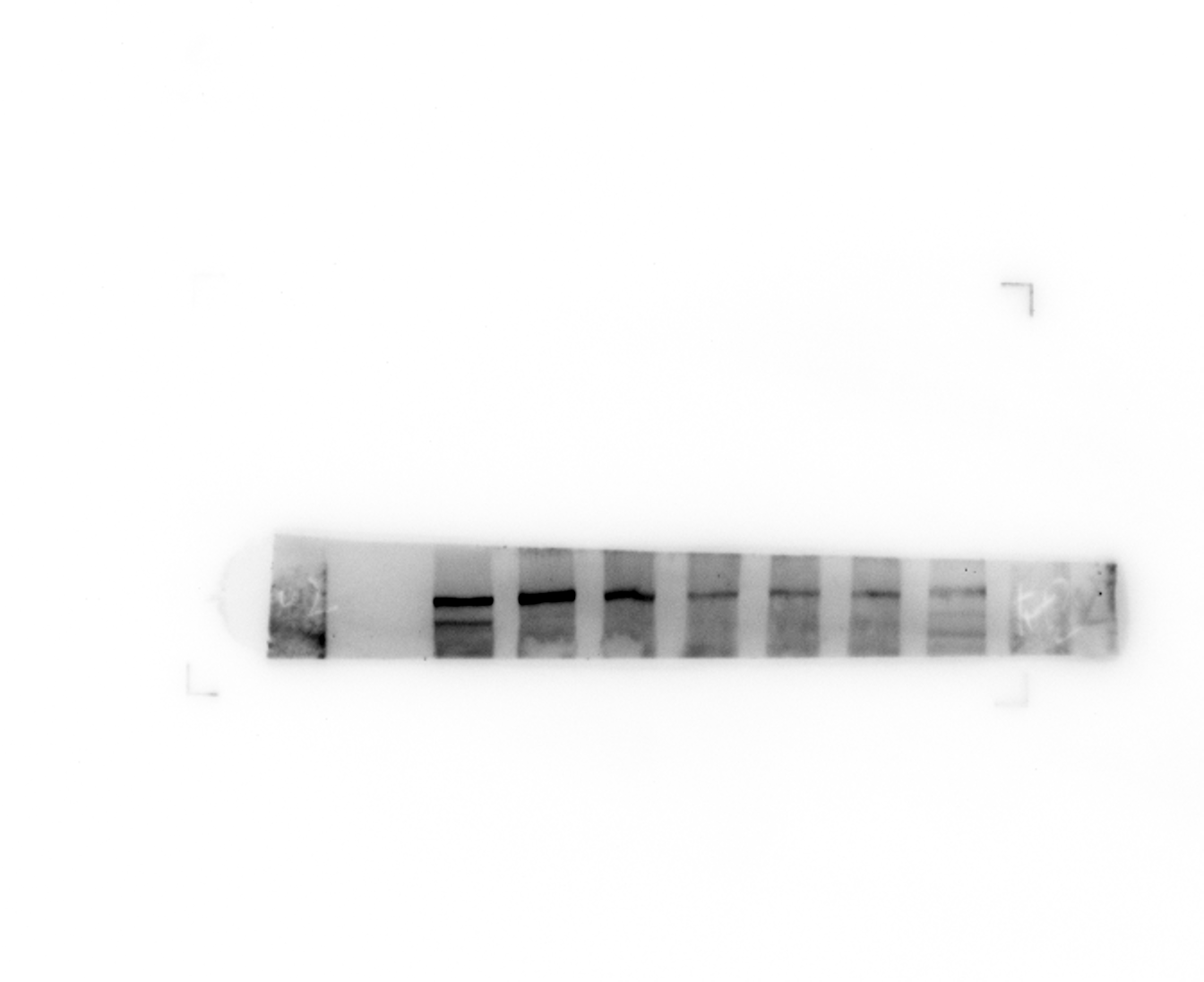

Supplement: Supplementary file 1 [file DataSheet1.ZIP › original source data/FIGERE 9 Anticancer effect of fluoxetine in A549 cells/Wesrern blotting of A549 cell/9h2 cleaved-caspase5.Tif]

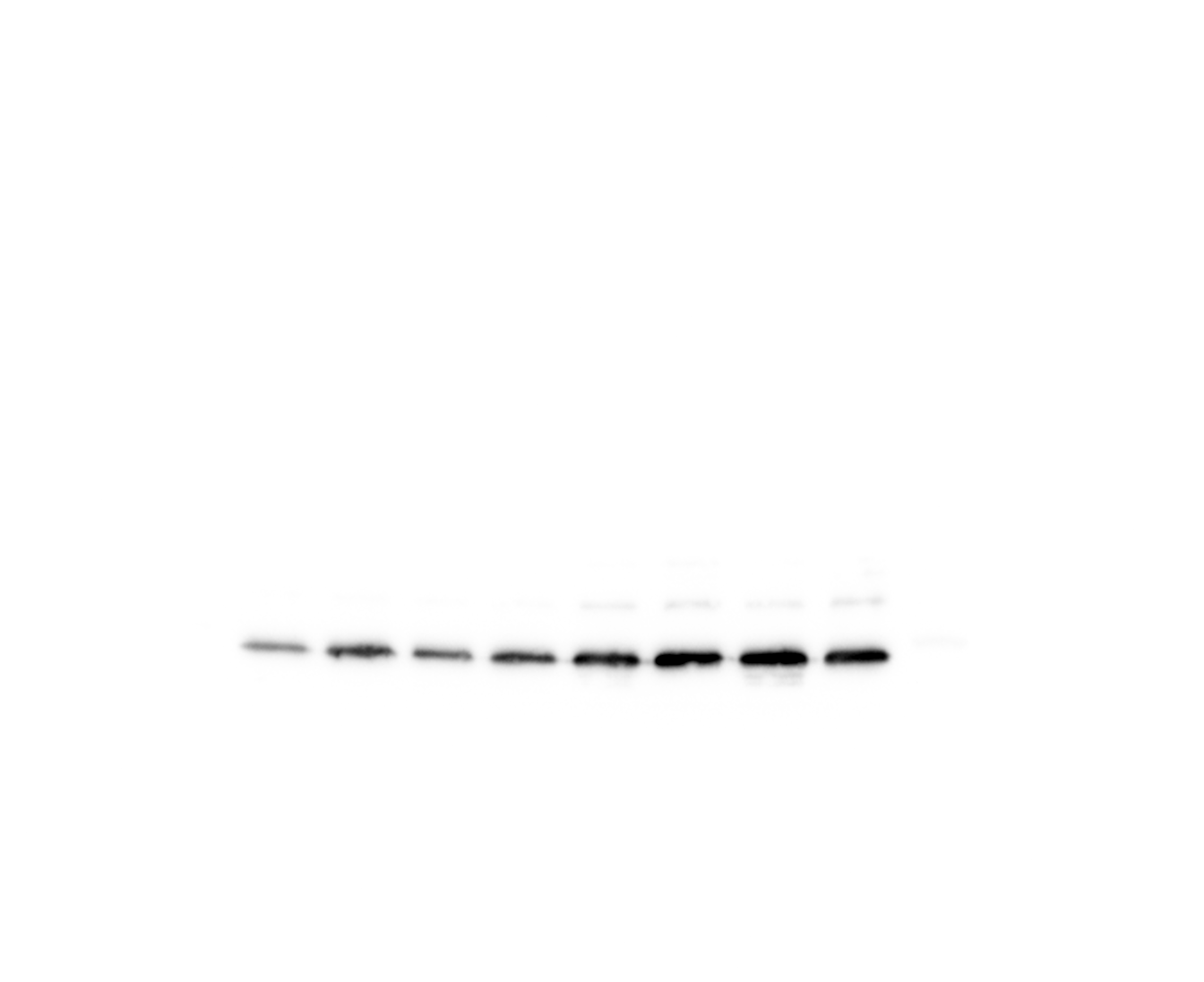

Supplement: Supplementary file 1 [file DataSheet1.ZIP › original source data/FIGERE 9 Anticancer effect of fluoxetine in A549 cells/Wesrern blotting of A549 cell/9h3 cleaved-caspase4.Tif]

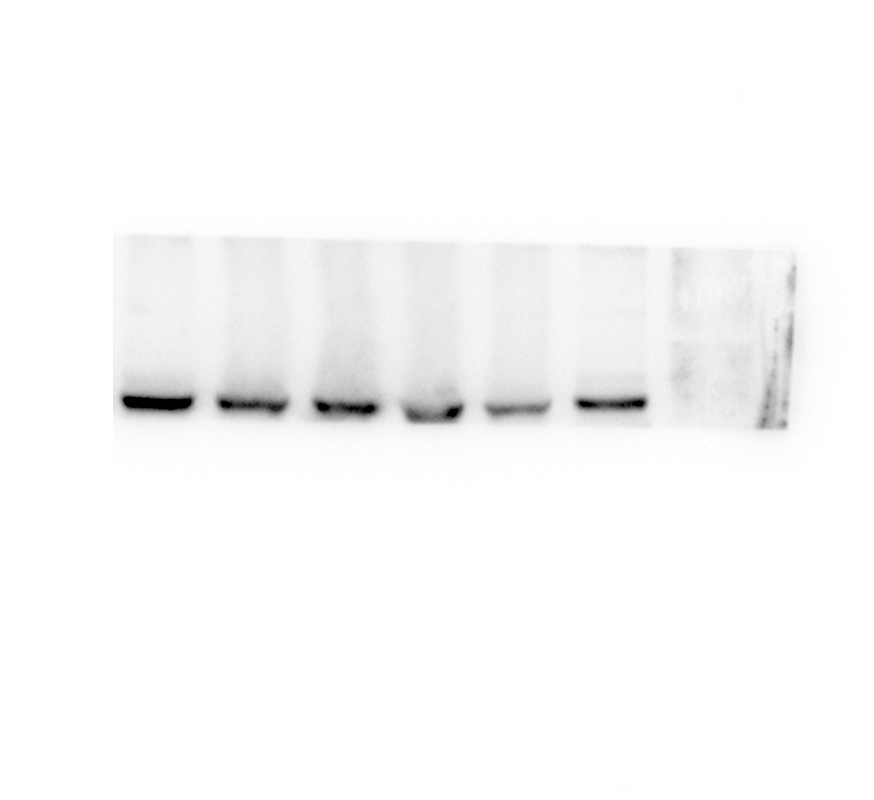

Supplement: Supplementary file 1 [file DataSheet1.ZIP › original source data/FIGERE 9 Anticancer effect of fluoxetine in A549 cells/Wesrern blotting of A549 cell/9h4 cleaved-caspase3.tif]

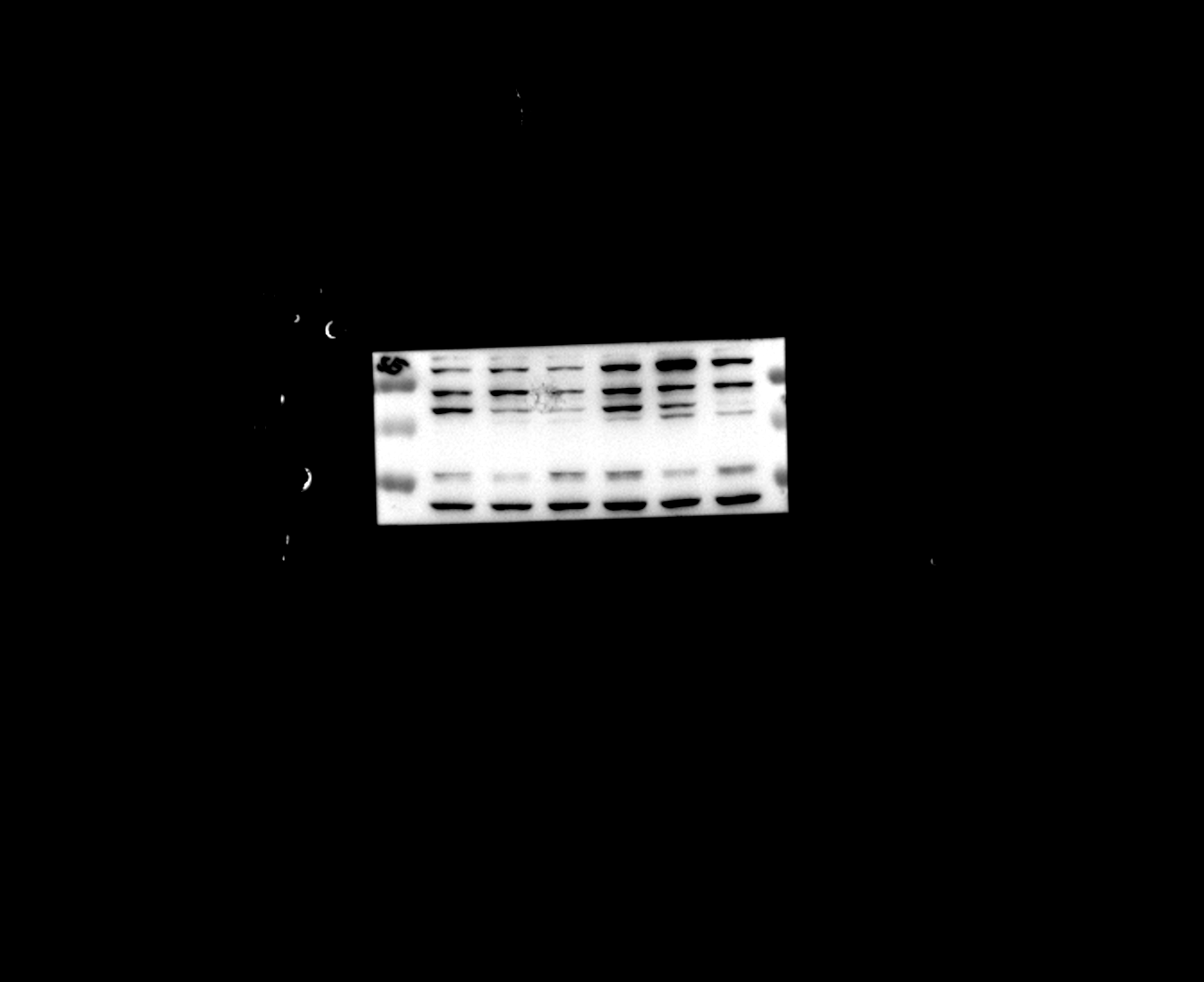

Supplement: Supplementary file 1 [file DataSheet1.ZIP › original source data/FIGERE 9 Anticancer effect of fluoxetine in A549 cells/Wesrern blotting of A549 cell/9h5 cleaved-caspase1.Tif]

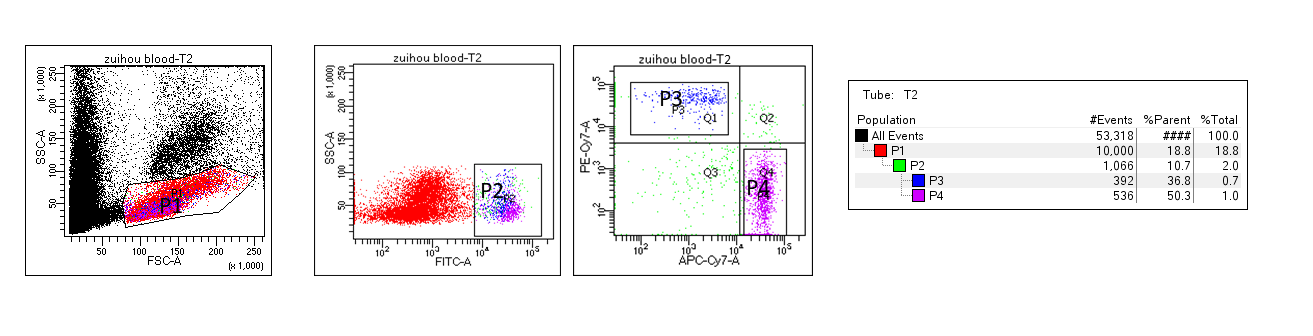

Supplement: Supplementary file 1 [file DataSheet1.ZIP › original source data/Flow Cytometry Analysis/A.tif]

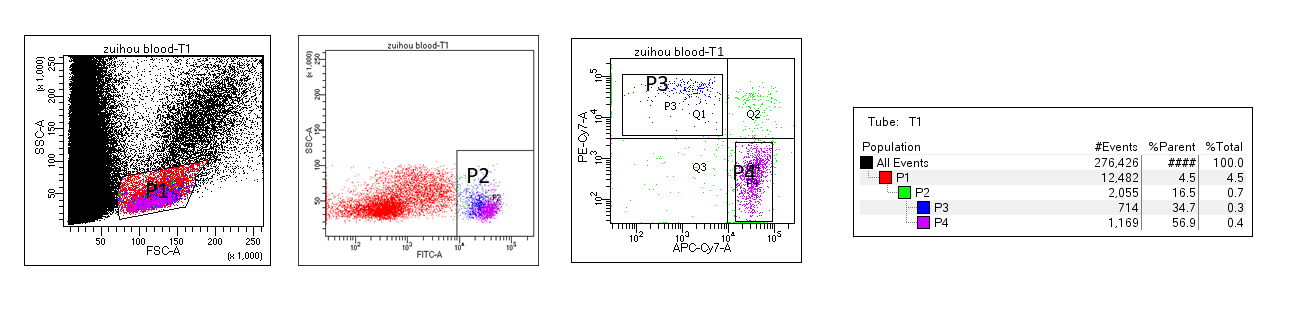

Supplement: Supplementary file 1 [file DataSheet1.ZIP › original source data/Flow Cytometry Analysis/B.tif]

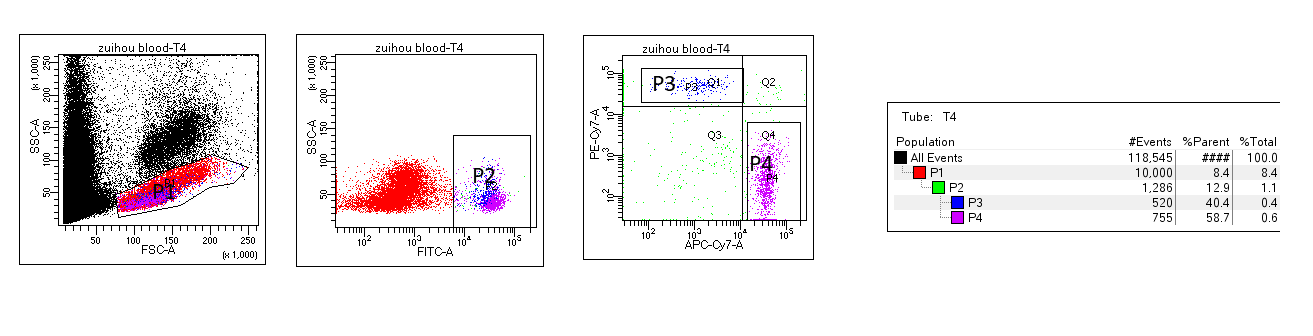

Supplement: Supplementary file 1 [file DataSheet1.ZIP › original source data/Flow Cytometry Analysis/C.tif]

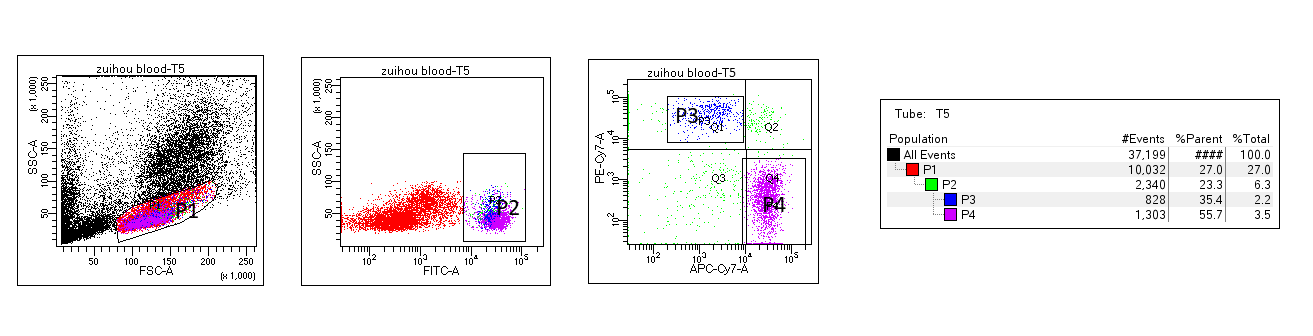

Supplement: Supplementary file 1 [file DataSheet1.ZIP › original source data/Flow Cytometry Analysis/D.tif]

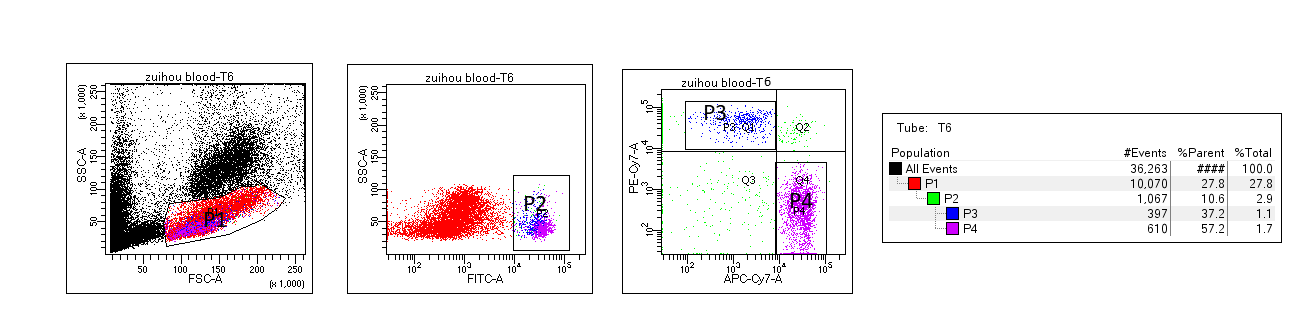

Supplement: Supplementary file 1 [file DataSheet1.ZIP › original source data/Flow Cytometry Analysis/E.tif]

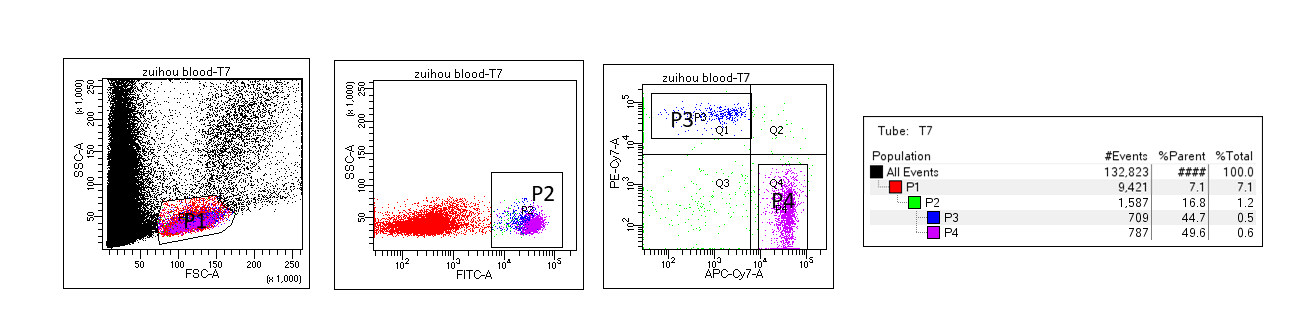

Supplement: Supplementary file 1 [file DataSheet1.ZIP › original source data/Flow Cytometry Analysis/F.tif]

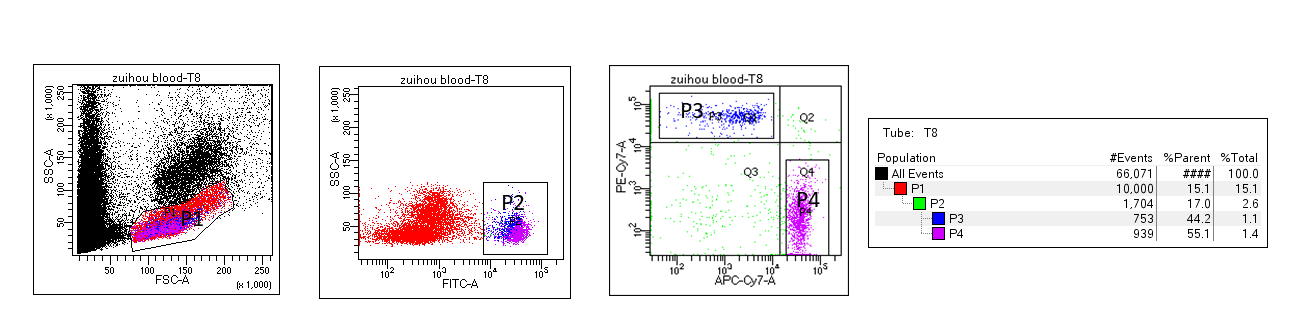

Supplement: Supplementary file 1 [file DataSheet1.ZIP › original source data/Flow Cytometry Analysis/G.tif]
